# Procoagulant Extracellular Vesicles Increase Neuronal Tau expression, Metabolism and Processing Through Tissue Factor and Protease Activated Receptor 2

**DOI:** 10.1007/s10571-025-01658-7

**Published:** 2026-01-06

**Authors:** Sophie J. Featherby, Eamon C. Faulkner, Andrew Gordon, Camille Ettelaie

**Affiliations:** Centre for Biomedicine, Hull-York Medical School, Cottingham Road, Hull, HU6 7RX UK

**Keywords:** Tissue factor, Tau, Phosphorylation, Neuronal cells, Neuropathies, Protease activated receptor 2, Factor VIIa, Extracellular vesicles, Proteolytic digestion, Aggregation

## Abstract

Neuro-inflammation is implicated in the onset of neuropathologies and can be promoted by stroke, trauma, toxins or infections. Brain tissue is rich in Tissue factor (TF) which is also released within cerebrospinal fluid as extracellular vesicles (EV). TF is an inflammatory protein which is increased during chronic conditions, and initiates blood coagulation and promotes tissue repair. This study examined the influence of TF on the expression, phosphorylation, aggregation and degradation of Tau protein in differentiated human cells SH-SY5Y and HCN-2, and rat neuronal cells. Studies were performed using vesicles containing TF or recombinant TF supplemented with factor VIIa (fVIIa) and also in the presence of various reagents and antibodies. Treatment of the differentiated cells with TF or TF-EV, upregulated the expression of Tau mRNA and protein, and was enhanced on repeated treatment. Incubation of cells with TF-fVIIa increased Tau expression and resulted in significant phosphorylation at Thr181, and was less at Ser202. Inhibition of the protease activity of TF-fVIIa, or blocking PAR2 activation on cells using SAM11 antibody, reduced Tau phosphorylation at Thr181. Examination of the Tau protein at intervals post-treatment indicated that Thr181 phosphorylation was present in bands of approximately 50 and 30–35 kDa while phosphorylation of Ser202 was associated with a 43 kDa band. Exposure of the cells to TF alone was sufficient to induce PKC-dependent phosphorylation of Tau. Prolonged treatment of differentiated SH-SY5Y cells with TF, resulted in higher staining with Amytracker dye. Finally, controlled digestion of recombinant full-length Tau with TF-fVIIa resulted in a smaller fragment. In conclusion, our data presents potential mechanisms by which TF influences Tau metabolism in neurons, being both beneficial in terms of clearance and regeneration, and having detrimental outcomes including aggregation.

**Supplementary Information:**

The online version contains supplementary material available at 10.1007/s10571-025-01658-7.

## Background

The association between chronic inflammation and neurodegenerative conditions has been established and include Alzheimer’s disease, Parkinson’s disease, chronic traumatic encephalopathy and multiple sclerosis, and several links with the progression of the chronic disease have been reported (Kinney et al. 2018; Walters et al. 2016; Sudduth et al. 2013; Attiq et al. 2025; Cheng et al. 2020; Coclitu et al. 2025; Demirkiran et al. 2021; Doroszkiewicz et al. 2025; Folta et al. 2025; Gomez-Nicola and Boche 2015; Kirsch et al. 2023; Knezevic and Mizrahi 2018; Lagarde et al. 2018; Liu et al. 2025; Seplovich et al. 2025; Shi and Yong 2025; Verma et al. 2025; Zedde et al. 2025). Neuro-inflammation has been attributed to the breakdown of the balance of pro- and anti-inflammatory mediators particularly, involving the activation of microglia in response to infection, toxins and injury. The activation of microglia is fundamental in the pro-inflammatory responses that lead to the formation of the plaques. However, the appearance of neurofibrillary tangles (NFT) may precede the onset of the disease in patients and in animal models (Guillozet et al. 2003; Nelson et al. 2009, 2012) and cerebral plaques are reported to form a decade or more before the appearance of disease symptoms, which lead to diagnosis (Bateman et al. 2012; Holper et al. 2022; Trojanowski et al. 2010). The association between Tau (microtubule associated protein 4) and proteopathies have been established (Holper et al. 2022; Ost et al. 2006; Taghdiri et al. 2019) and Tau expression, phosphorylation, digestion and aggregation have been implicated in the neuronal degeneration (Arai et al. 2005; Basheer et al. 2023; Fourest-Lieuvin et al. 2023; Kopke et al. 1993; Meredith et al. 2013; Neddens et al. 2018; Rawat et al. 2022; Santpere et al. 2006; Sattarov et al. 2024; Stefanoska et al. 2022; Takeda et al. 2015; Tenreiro et al. 2014; Wagshal et al. 2015; Wang et al. 2007, 2009; Wegmann et al. 2021). However, there appears to be a gap in the identification of the early factors that initiate the pathology. One contributor to the pathogenesis of neuropathies is trauma to the brain, for example through major injury or repeated impacts (Bielanin et al. 2024; Cheng et al. 2020; Jacquens et al. 2024; Kirsch et al. 2023; Miner et al. 2024; Ost et al. 2006; Phelps et al. 2020; Zetterberg et al. 2006). The brain is highly dependent on the extrinsic coagulation pathway to stop haemorrhages. The extrinsic pathway of coagulation is initiated by a 47 kDa glycoprotein named “Tissue Factor (TF)” also known as coagulation factor III (Bachli 2000; Carson and Konigsberg 1980; Kirchhofer and Nemerson 1996; Nemerson and Repke 1985). TF is exposed upon injury or trauma to the cells surrounding the blood system and initiates blood coagulation by acting as a cofactor that activates coagulation factor VII, which in turn activates two other blood coagulation proteins, factor X and IX (Carson and Konigsberg 1980; Kirchhofer and Nemerson 1996; Nemerson and Repke 1985). TF is expressed by neurons, astrocytes and microglial cells, and TF concentrations are some of the highest in the brain (Carson et al. 1987; Drake et al. 1989; Faulk et al. 1990; Fleck et al. 1990; Gonzalez-Dunia et al. 1996; Grover and Mackman 2018; Wang et al. 2016). TF appears to have a central role connecting blood coagulation and inflammation (Mackman 2009; Wilhelm et al. 2023; Zelaya et al. 2018). Stimulation of cells with inflammatory cytokines, endotoxins, hypoxia and oxidative stress, or activation of protease activated receptor (PAR) 2 can induce the expression and release of TF as procoagulant extracellular vesicles (EV) (Ansari et al. 2021; Bastarache et al. 2011; Collier and Ettelaie 2011; Ettelaie et al. 2013, 2022; Macey et al. 2010; Marutsuka et al. 2002; Nielsen et al. 2016; Ovstebo et al. 2012; Rothmeier et al. 2019; Sachetto and Mackman 2023; Schecter et al. 2000; Siegbahn et al. 2008; Svensson et al. 2011; Szotowski et al. 2005; Yokota et al. 2009). These TF-positive EV raise the risk of thrombosis as well as inducing cell signalling resulting in alterations in cells (Hisada and Mackman 2019; Li et al. 2022; Rondon et al. 2019). Elevated levels of TF have been reported in a few neuropathies (Leung et al. 2015; Maegele et al. 2020; McComb et al. 1991; Ziliotto et al. 2021; Zimmermann et al. 2024) and TF has also been reported to influence behaviour (Doulalas et al. 2006; Hedner 1998; Yang et al. 2016). TF has a diffuse expression in normal brain tissue but localises at the corona of the senile plaques in Alzheimer’s tissue (McComb et al. 1991). Similarly, TF is elevated in the cerebrospinal fluid from patients with Parkinson’s disease (Leung et al. 2015). However, neither of these are direct evidence for the alterations in TF expression or activity, or the evidence that TF has a direct outcome on neuropathies. The exposure of TF at the site of injury or inflammation also initiates signalling mechanisms that lead to repair, or alternatively removal of injured cells (Featherby et al. 2025a). These processes are mediated by both protease-independent (Ettelaie et al. 2007; Featherby et al. 2025b; van den Hengel and Versteeg 2011) and dependent mechanisms (Camerer et al. 2000; Ettelaie et al. 2007; Kamath et al. 2001). Some of these mechanisms appear to be common in promoting the release of both TF and Tau (Clark et al. 1991; Masliah et al. 1990). In this study, the outcome of treatment of neuronal cells with TF, in the presence and absence of factor VIIa was explored to emulate increased inflammation, or bleeding in the brain. The expression and phosphorylation of Tau was examined in separate neuronal cells and the degradation and aggregation of the Tau explored.

## Materials and Methods

### Cell Culture, Differentiation and Analysis

SH-SY5Y human neuroblastoma cell line (ECACC, Salisbury, UK; Supplementary Fig. 1 A) were propagated in DMEM containing 10% (v/v) foetal calf serum (FCS) and differentiated using Brain-Derived Neruonal Factor (BDNF; 20 ng/ml; Qkine, Cambridge, UK) and retinoic acid (10 µM; Sigma Chemical Co. Ltd, Poole, UK) with progressive reduction of FCS, over 10 days (Shipley et al. 2016; Targett et al. 2024). HCN-2 human cortical neuron cell line (LGC-ATCC, Teddington, UK; Supplementary Fig. 1B) were cultured in low-bicarbonate-DMEM (LGC-ATCC), containing 12% (v/v) FCS, and differentiated using Nerve Growth Factor protein (NGF; 25 ng/ml; Stratech, Cambridge, UK), Bucladesine/dcAMP (0.5 mM; Stratech) and 3-Isobutyl-1-methylxanthine (0.5 mM; Sigma Chemical Co Ltd, Poole, UK) over 10 days (Ronnett et al. 1994). Rat cortex brain cells (Thermo Fisher Scientific, Warrington, UK) were cultured in complete B-27 Plus Neuronal Culture Medium. Differentiation of the cells to neuronal morphology were confirmed by examining the expression of combinations of neuronal markers; Tubulin β-III, Microtubule associated protein 2 (MAP2), Neurofilament light chain (NF-L), Synaptophysin, Doublecortin, γ-Enolase (NSE), as well as Neurogenic differentiation 1 (NeuroD1) Ser274 phosphorylation. The levels of the neuro-markers were examined by western blot, and were probed using rabbit anti-human antibodies for the above markers (Abbexa Ltd, Cambridge, UK), as outlined below (for a complete list of antibodies see Supplementary Table 1).

### Preparation of TF-EV and Treatment of Neuronal Cells

Extracellular vesicles were isolated from human dermal blood microvascular endothelial cells (PromoCell, Heidelberg, Germany) propagated in EC-MV media containing 5% (v/v) FCS and growth supplements (PromoCell). The cells were transfected with the pCMV-Ac-TF-tGFP plasmid or pCMV-Ac-tGFP plasmid (OriGene. Rockville, USA) to express TF-tGFP, or tGFP alone. Endothelial cells were chosen since these cells do not express endogenous TF (Madkhali et al. 2021). In addition to TF, these vesicles were shown to contain additional proteins including some fVII/fVIIa (Madkhali et al. 2021). All procedures for the transfection of endothelial cells, induction of protein expression, adaption of cells to serum-free medium, cell activation and the isolation and characterisation of TF-containing EV and control-EV were as described in detail and verified previously (Ettelaie et al. 2014; Featherby and Ettelaie 2024). Alternatively, cells (2 × 10^5^) were incubated with recombinant relipidated Innovin TF (stock = 0.13 µg/ml = 1000 U/ml; Dade Behring, Inc.) with or without human fVIIa (5 nM; Enzyme Research Lab., Swansea, UK). The relipidated TF contains the recombinant protein reconstituted in vesicles of known lipid composition and is therefore a substitute for extracellular vesicles but devoid of additional proteins.

In some experiments, the TF aliquots were pre-incubated for 1 h, with a mouse anti-human-TF antibody, 10H10 (20 µg/ml; BD Bioscience, Wokingham, UK) capable of blocking TF signalling, a mouse anti-human-TF antibody, HTF-1 (20 µg/ml; eBioscience/Thermo Scientific) to block TF-fVIIa protease/procoagulant activity, or a mouse control isotype IgG antibodies (20 µg/ml; New England Biolabs, Hitchin, UK). In other experiments, fVIIa was pre-incubated for 1 h with the chemical inhibitor PCI27483 (Caymen Chemical Co./Cambridge Bioscience, Cambridge, UK). Alternatively, the neuronal cells were treated with a rat anti-human antibody (20 µg/ml; AIIB2; Merck KGaA) to block β1-integrin signalling, a mouse anti-human PAR2 antibody, SAM11 (20 µg/ml; Santa Cruz Biotechnology, Heidelberg, Germany), capable of blocking PAR2 signalling, or PAR2-activating peptide (PAR2-AP; 20 µM) to induce PAR2 signalling.

### Western Blot Analysis

Cells (2 × 10^5^) were lysed in cell lysis reagent (Promega Corporation Inc. Southampton, UK) at 4˚C for 30 min on a rotator. The protein content of samples was assessed using Pierce BCA protein assay (Thermo Fisher Scientific, Inc.) in accordance with the manufacturer’s instructions. Samples were added to Laemmli buffer (Sigma-Aldrich; Merck KGaA; solution contains 4% SDS, 20% glycerol, 10% 2-mercaptoethanol, 0.004% bromophenol blue and 0.125 M Tris HCl; pH 6.8) and heated to 98 °C for 10 min. Aliquots (10 µg protein) of the lysates were separated by electrophoresis carried out on a denaturing 12% (w/v) polyacrylamide gel (Flowgen, Nottingham, UK). The order of sample loading was altered to prevent any anomalies from loading bias. The separated proteins were then transferred to a nitrocellulose membrane (GE Healthcare) and blocked with Tris-buffered saline Tween 0.01% (v/v) (TBST; Sigma-Aldrich; Merck KGaA; pH 8) containing 1% (w/v) Bovine Serum Albumin (BSA), at room temperature for 60 min. The membranes were probed overnight at 4˚C with the appropriate antibodies diluted 1:3000 (v/v) in TBST. Membranes were then washed and developed at room temperature for 60 min with goat anti-rabbit IgG alkaline phosphatase-conjugated antibody or goat anti-mouse IgG alkaline phosphatase-conjugated antibody (Santa Cruz Biotechnology, Inc.) as appropriate, diluted 1:3,000 (v/v) in TBST, and visualised using the Western Blue stabilised alkaline phosphatase-substrate (Promega Corporation). Antibodies used were rabbit anti-human Tau antibodies (Proteintech, Manchester, UK and Abbexa Ltd, Cambridge, UK) and mouse anti-human Tau antibody (BioLegend, London, UK) and rabbit anti-human phospho-Thr181 Tau (Proteintech, and Abbexa Ltd), rabbit anti-human phospho-Ser202 Tau, rabbit anti-human phospho-Thr217 Tau or rabbit anti-human phospho-Ser396 Tau (Abbexa Ltd). To detect the expression and the activation state of fVIIa, membranes were probed with a polyclonal rabbit anti human factor VII antibody, capable of detecting both the intact fVII and the chains of the activated fVIIa (Abcam, Cambridge, UK). Other antibodies were used to confirm cell differentiation as listed above. All measurements were normalised against the respective Ubiquitin Carboxy Terminal Hydrolase L1, Tubulin β (Abbexa Ltd) probed with respective rabbit anti-human antibodies (Abbexa Ltd) and/or GAPDH (V18; Santa Cruz Biotechnology). All antibodies were diluted 1:3,000 (v/v) in TBST. Membranes were then washed and developed at room temperature for 60 min with goat anti-rabbit IgG alkaline phosphatase-conjugated antibody, or donkey anti-goat IgG alkaline phosphatase-conjugated antibody (Santa Cruz Biotechnology, Inc.), diluted 1:3,000 (v/v) in TBST, and visualised using the Western Blue stabilised alkaline phosphatase-substrate. The band densities were analysed using the ImageJ 1.53t Software (National Institutes of Health).

In some experiments the cells were treated with TF (0.65 ng/ml) and supplemented with a second dose after 48 h and the conditioned media was collected and centrifuged at 3000 *g* for 5 min to remove cell debris. The media were then added to Centricon concentrators with 3 kDa cutoff (Amicon, Beverly, USA) and centrifuged at 3000 *g* for 5 h, at 4 °C, to concentrate the proteins. The retained proteins were then separated by 12% (w/v) SDS-PAGE, transferred to nitrocellulose membranes and probed for Tau, or phospho-Thr181 Tau using antibodies as above.

### Immunoprecipitation of Tau and Analysis of Phosphorylation by PKCα

Cells (2 × 10^5^) were incubated with TF (0.65 ng/ml) in the presence or absence of fVII (5 nM) for 24 h. Additionally, to block the phosphorylation of Tau by PKC, cells were pre-incubated with the PKC inhibitor Gö6976 (100 nM; R&D Systems, Abingdon, UK) for 40 min, prior to addition of TF. Cells were then lysed in 150 µl of Phosphosafe lysis buffer (Merck-Millipore, Nottingham, UK) containing a protease inhibitor cocktail (1% v/v) (Sigma Chemical Company Ltd, Poole, UK). Tau protein was immunoprecipitated from cell lysates using a mouse anti-human Tau monoclonal antibody (210–230; 2 µg per sample; BioLegend) alongside an IgG isotype (Cell Signalling Technology). All samples were incubated at 4 °C overnight with gentle shaking. Pureproteome protein A-magnetic beads (10 µl) (Merck-Millipore) was added to all samples and controls and incubated at 4 °C for 90 min with shaking. The samples were then placed in a magnetic stand and the supernatant removed, washed three times (1 ml) with PBS-Tween 20 (0.1% w/v) and the samples denatured in SDS-PAGE loading buffer (50 µl) (Sigma). The samples were then examined by western blot using a rabbit anti-phospho-PKC-substrate motif antibody (Cell Signalling Technology) diluted 1:2000 (v/v) in TBST buffer as previously described (Collier and Ettelaie 2011).

### Analysis of the Digestion of Recombinant Tau Protein by TF-fVIIa Complex

Recombinant full-length His tag-Tau (Tau-441; 40 µg/ml; Sino Biologicals/Stratech) with the His tag on the N-terminal of the Tau protein, was digested with TF-fVIIa complex. Reactions were prepared to include recombinant TF (1.3 ng/ml) and fVIIa (10 nM), Tris-HCl pH 7.4 (5 mM) and CaCl_2_ (5 mM) and incubated at 37 °C for 1 h. In some reactions, the recombinant TF was pre-incubated with HTF-1 antibody (20 µg/ml) which inhibits the protease function of TF-fVIIa, or by inclusion of fVIIa inhibitor, PCI27483 (10 µg/ml). Other reactions were prepared in the presence of fVIIa alone, or fXa (10 nM; Enzyme Research Lab). Separate sets of the proteins were then separated by SDS-PAGE and probed by western blot as above, using an alkaline phosphates-conjugated anti-His-Tag antibody (1:3000 v/v; Santa Cruz Biotechnology). The bands were visualised using the Western blue substrate (Promega) and the molecular weight of the bands were estimated using ImageJ program. Other sets were probed using two different rabbit anti-human Tau polyclonal antibodies obtained from Proteintech and Abbexa (diluted 1:3000 v/v in TBST), and a mouse anti-human Tau monoclonal antibody (210–230; BioLegend; diluted 1:3000 v/v in TBST). The membranes were then developed with an alkaline phosphatase conjugated goat anti-rabbit antibody or goat anti-mouse antibody, diluted 1:3000 (v/v) and visualised using the Western Blue stabilised alkaline phosphatase-substrate (Promega) and recorded.

### White Light and Fluorescence Microscopy

Cells (2 × 10^5^) were treated with recombinant TF and fVIIa as stated above, fixed with 4% (v/v) paraformaldehyde for 15 min at room temperature, and then washed 3 times with PBS. The axonal and dendritic outgrowth and connectivity was monitored using a Nikon TMS trinocular inverted phase contrast microscope and images were acquired using a Nikon Coolpix 5000 camera at room temperature, using a stage micrometre. To detect the formation of protein aggregates, SH-SY5Y cells (5 × 10^4^) were plated in 29 mm culture dishes with a 10 mm glass bottomed micro-well (InVitro Scientific/Cellvis, Sunnyvale, USA) and differentiated as above. The cells were treated daily with TF (0.65 ng/ml) in the presence and absence of fVIIa (5 nM) for up to 3 days. The cells were then fixed, washed and probed with Amytracker 630 (Ebba Biotech AB, Solna, Sweden) diluted 1 µg/ml in water. The cells were stained with DAPI (2 µg/ml; Sigma) and Phalloidin-iFluor 488 (2 µg/ml; Abcam) and images were acquired at room temperature, using a Zeiss Axio Vert.A1 inverted fluorescence microscope (Carl Zeiss Ltd, Welwyn Garden City, UK) at × 40 magnification. Images were acquired using the ZEN software (Carl Zeiss Ltd) and the filters were selected for DAPI, FITC and Texas Red.

### RNA Isolation and RT-PCR

Total RNA was isolated using the Monarch total RNA extraction kit (New England BioLabs, Inc.) from 10^5^ cells. Samples of the extracted RNA (100 ng) were amplified using the QuantiTect primer set specific for human Tau mRNA (Qiagen-UK, Manchester, UK; sequence not disclosed by the company). The relative amount of each mRNA was determined against β-actin using QuantiTect primer set (Qiagen-UK). GoTaq 1-Step RT-qPCR System contained GoScript Reverse Transcriptase and RNasin Plus RNase Inhibitor. RT was performed at 48˚C for 30 min. The GoTaq 1-Step RT-qPCR System also contained GoTaq Hot Start Polymerase, BRYT Green fluorescent dye, MgCl_2_, dNTPs and a proprietary reaction buffer. The qPCR reactions consisted of a denaturing step at 95˚C for 15 s and a combined annealing and extending step at 60˚C for 1 min. The reactions were performed using an iCycler thermal cycler (Bio-Rad Laboratories, Inc., Hemel Hempstead, UK) for 40 cycles. Following amplification, the relative amounts of target mRNA were determined using the 2^−ΔΔCq^ method (Livak and Schmittgen 2001).

### Data Calculation and Statistical Analysis

All measurements from the western blots were normalised against the respective GAPDH sample to ensure loading uniformity. The average of the non-treated samples in each section was then calculated, and all values were determined as the ratio of measurements against the average of the non-treated samples (indicated as “Ratio vs the average of non-treated). Where appropriate, the ratio of phospho-Tau to total Tau protein was also calculated and included. Presented data include the calculated mean values ± the calculated standard deviation. The number of experiments is stated in the legend and in experiments where the number of repeats exceeded 4, also included in each column. Statistical analysis was carried out using the GraphPad Prism version 10.5 (GraphPad Software, Boston, Massachusetts USA). Data were analysed for normality using Shapiro-Wilk test. Significance was determined using one-way ANOVA (analysis of variance) and Tukey’s honesty significance test. Following the first few experiments, the required sample size was calculated to produce the highest level of confidence (Kadam and Bhalerao 2010) (σ = 0.29; Δ = 1.1, after 1 day treatment).

## Results

### Confirmation of the Differentiation Status of Cells

**Fig. 1:**
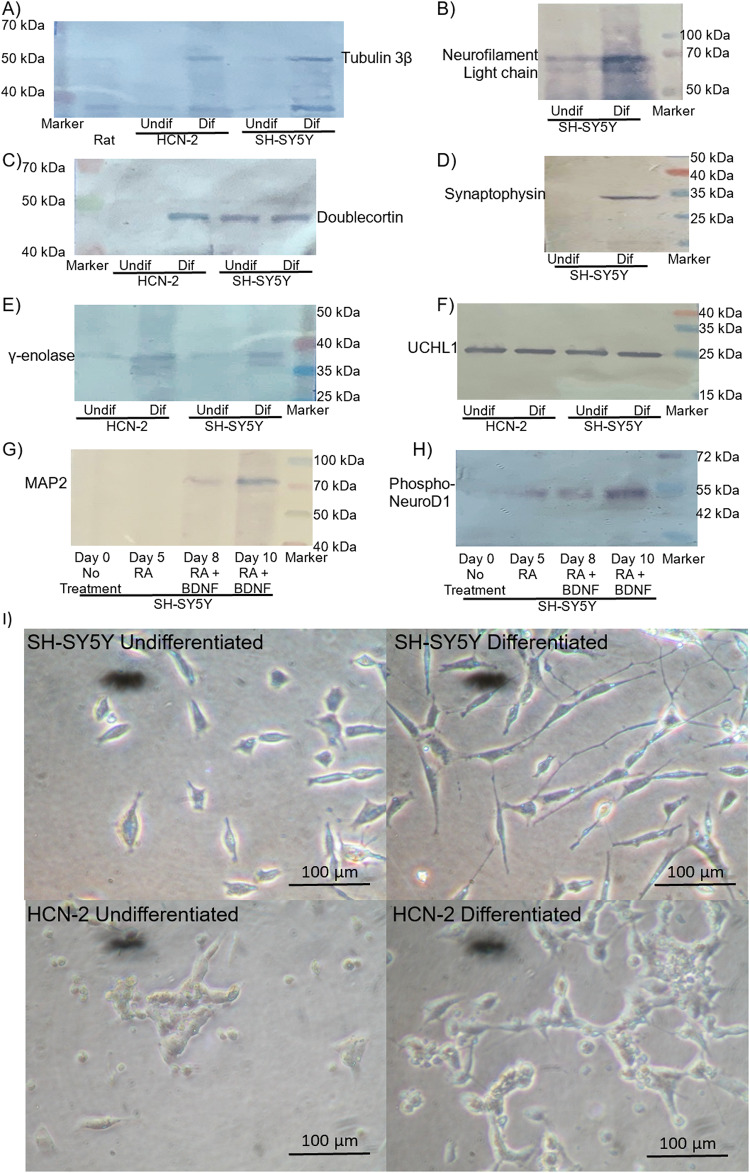
Confirmation of the differentiation of SH-SY5Y and HCN-2 cells. SH-SY5Y human neuroblastoma cells were differentiated using Brain-Derived Neuronal Factor (BDNF; 20 ng/ml) and retinoic acid (RA; 10 µM) with progressive reduction of FCS, over 10 days. HCN-2 human cortical neuron cells were differentiated using Nerve Growth Factor protein (NGF; 25 ng/ml), Bucladesine/dcAMP (0.5 mM) and 3-Isobutyl-1-methylxanthine (0.5 mM) over 10 days. **A**–**E** Differentiation of the cells (2 × 10^5^) to neuronal morphology were confirmed by examining the expression of combinations of neuronal markers by western blot analysis; **A** Tubulin β-III, **C** Doublecortin and **E** γ-Enolase in both cell lines, and **B** Neurofilament light chain and **D** Synaptophysin in SH-SY5Y cells. As a control, the levels of **F** Ubiquitin Carboxyl Terminal Hyrdrolase L1 (UCHL1) were also determined. Additionally, **G** the levels of Microtubule associated protein 2 (MAP2) and **H** Neurogenic differentiation 1 (NeuroD1) phosphorylation (NSE) were monitored over the differentiation process. The lanes are labelled as undif = undifferentiated cells and dif = differentiated cells. **I** Cell morphology was recorded by light microscopy on a Nikon TMS microscope with a camera attachment. Size determination was performed using a stage micrometre. (Images are representative of 3 separate experiments)

HCN-2 and SH-SY5Y cells were differentiated according to previously published procedures (Ronnett et al. 1994; Shipley et al. 2016; Targett et al. 2024). To confirm the status of the cells prior to the studies, upregulation in the expression of several neuronal differentiation markers was examined; The complement of markers included Tubulin β-III, Neurofilament light chain (NF-L), Doublecortin, Synaptophysin, γ-Enolase (NSE), together with Ubiquitin Carboxyl Terminal Hyrdrolase L1 (UCHL1) (Fig. 1A-F). Additionally, the levels of Microtubule associated protein 2 (MAP2) and the phosphorylation of Ser274 on Neurogenic differentiation 1 (NeuroD1) were monitored over the period of differentiation in SH-SY5Y cells (Fig. 1G and H). The morphology of the cells was also recorded by white light microscopy (Fig. 1I) and indicated the presence of outgrowths in differentiated cells, although HCN-2 cells formed more dense clusters.

### Treatment of Cells with TF Upregulates the Expression of Tau and Induces Tau Phosphorylation

**Fig. 2:**
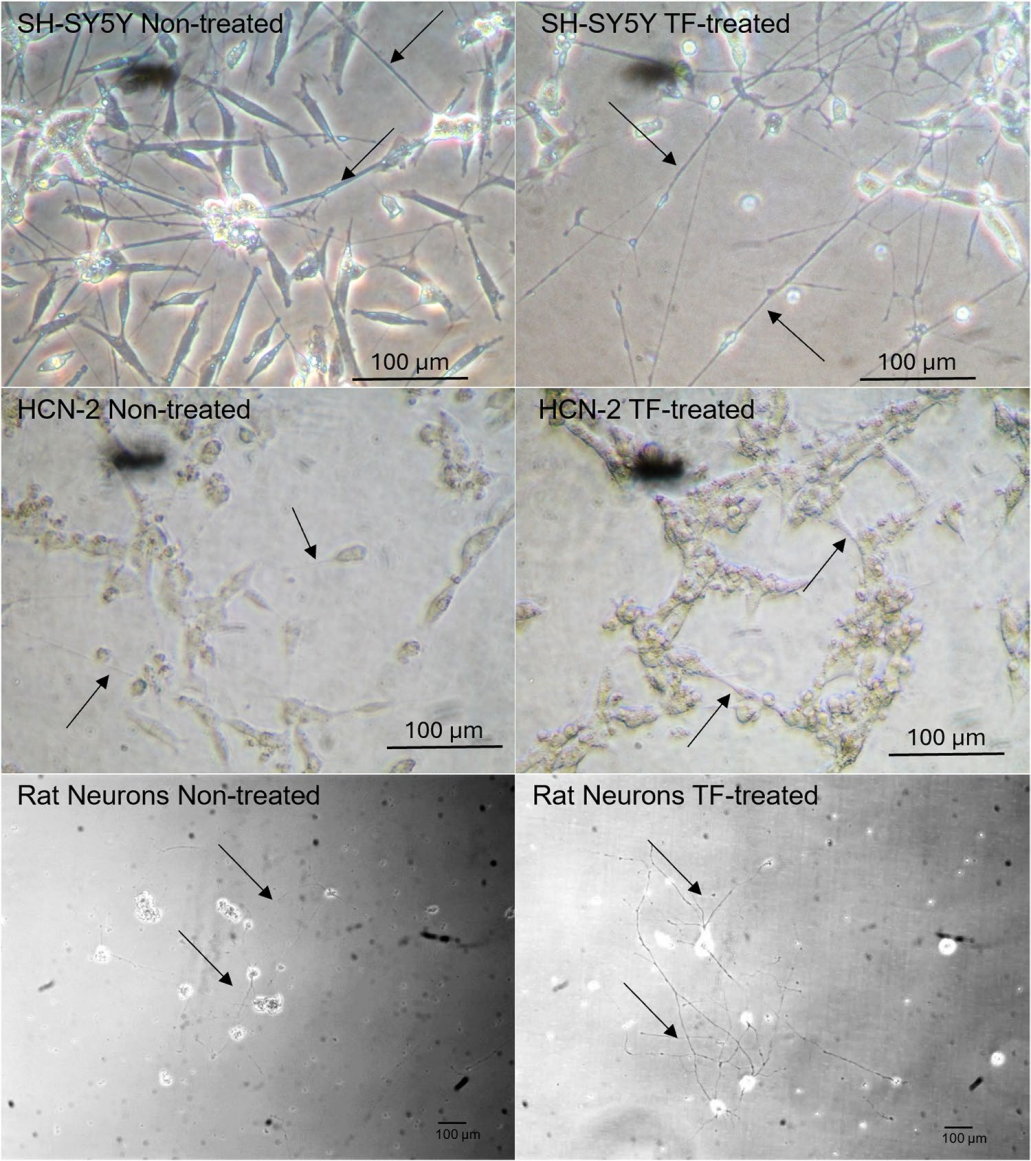
Examination of the influence of TF on differentiated SH-SY5Y, HCN-2 and rat neuronal cells. SH-SY5Y, HCN-2 and rat neuronal cells were plated in 29 mm culture dishes with a 10 mm glass bottomed micro-well and differentiated. Aliquots of the differentiated cells were treated daily with TF (0.65 ng/ml) and fVIIa (5 nM) for up to 3 days. Cellular outgrowth (indicated with arrows) and connectivity were monitored by light microscopy on a Nikon TMS microscope with a camera attachment. Size determination was performed using a stage micrometre and arrows indicate the neuronal outgrowth. (Images are representative of 3 separate experiments)

**Fig. 3:**
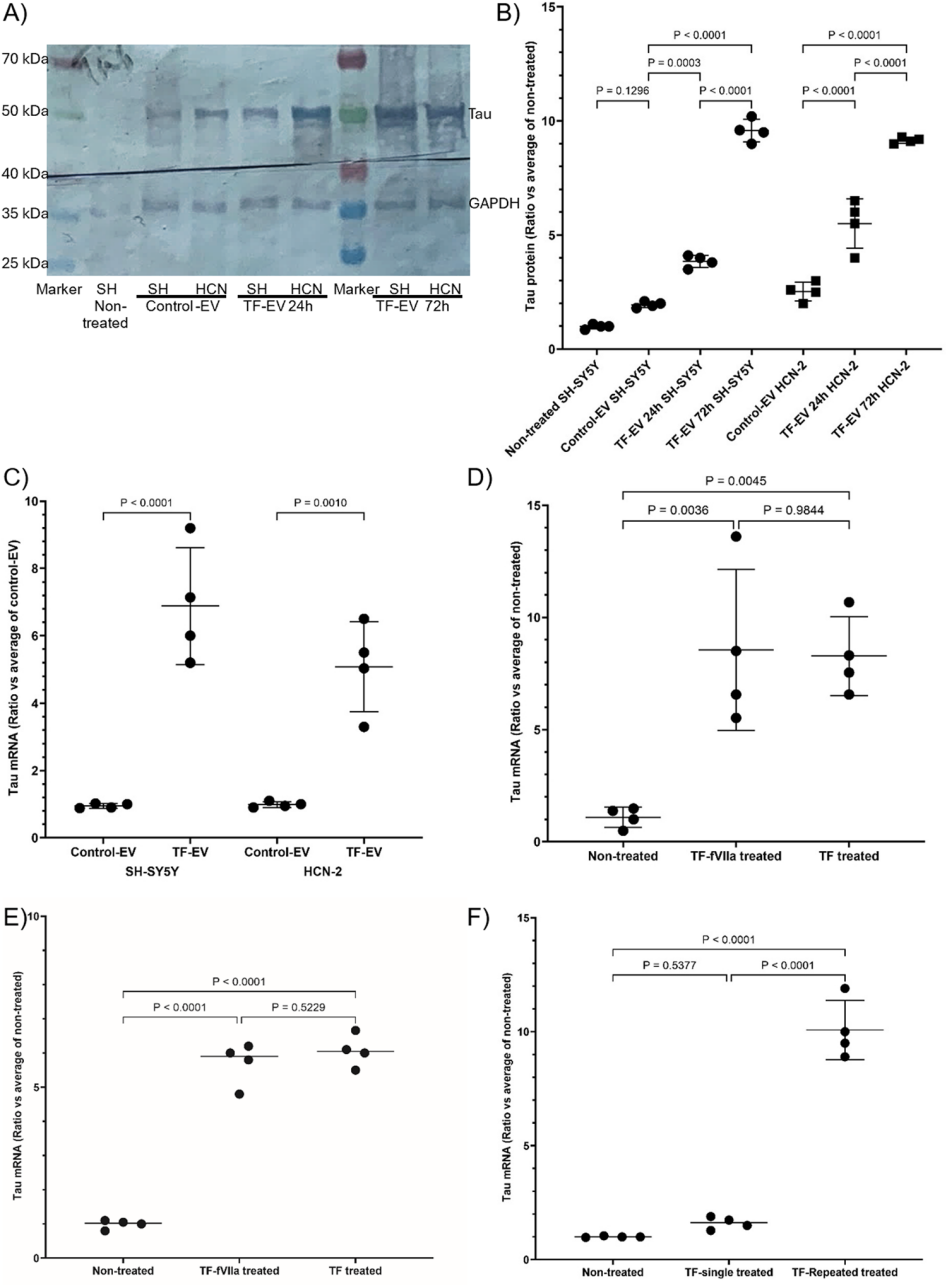
Analysis of the influence of TF-EV/recombinant TF on the expression of Tau protein and mRNA in differentiated SH-SY5Y and HCN-2 cells. Extracellular vesicles (EV) were isolated from primary endothelial cells transfected to express TF-tGFP, or tGFP alone. A) SH-SY5Y and HCN-2 cells (2 × 10^5^) were treated with TF-EV or control-EV. Sets of cells were harvested at 24 h and a second set were treated again and harvested at 72 h. **A** Cellular lysates (10 µg protein) were examined for Tau and phospho-Thr181 Tau by western blot analysis, and **B** band densities were determined. All values were normalised against the respective GAPDH and for comparison, all ratios were calculated against the average from the non-treated SH-SY5Y cells ± the calculated standard deviation. The data were obtained from 4 biological experiments, and all data groups were determined to have normal distributions. Additionally, SH-SY5Y and HCN-2 cells were treated with **C** TF-EV or control-EV and harvested at 24 h. Alternatively, **D** SH-SY5Y and **E** HCN-2 cells were treated with recombinant relipidated Innovin TF (0.65 ng/ml) with or without human fVIIa (5 nM) and harvested at 24 h. **F** Another set of HCN-2 cells were again supplemented with a second dose at 48 h, and harvested at 72 h. Total RNA was isolated and amplified using the QuantiTect primer for human Tau mRNA and the relative amount of each mRNA was determined against β-actin for 40 cycles. Following amplification, the relative amounts of target mRNA were determined using the 2^−ΔΔCq^ method. Presented data show the ratios, calculated against the average from the respective non-treated cells ± the calculated standard deviation. The data were obtained from 4 biological experiments, and all data groups were determined to have normal distributions

Incubation of cells with TF-fVIIa promoted greater density, and especially the average length of the outgrowth increased (Fig. 2 and Supplementary Table 2) and was reflected in increased expression of Doublecortin (Supplementary Fig. 2 A and B). Furthermore, increased expression of Synaptophysin (Supplementary Fig. 2 C and D) and PSD-95 (Supplementary Fig. 2E and F) were recorded, indicating synaptic formation. Additionally, the upregulation of Activity-regulated cytoskeleton-associated protein (Arc) indicated the increased synaptic plasticity in the treated cells (Supplementary Fig. 2G and H). Incubation of SH-SY5Y and HCN-2 cells with TF-tGFP containing EV isolated from endothelial cells resulted in increased expression levels of Tau protein compared to the control EV (Fig. 3A), doubling within 24 h, and over 4-fold by 72 h (Fig. 3B). The increase in Tau protein was also reflected in upregulation of Tau mRNA expression which were measured in SH-SY5Y and HCN-2 cells, only. The level of Tau mRNA increased by comparable levels in these cells on incubation with TF-EV (Fig. 3C) or recombinant TF (Fig. 3D and E) and was not significantly altered on inclusion of fVIIa. Moreover, repeated supplementation with recombinant TF over 72 h further enhanced Tau mRNA expression, while the levels returned to original levels without additional treatment (Fig. 3F).

**Fig. 4:**
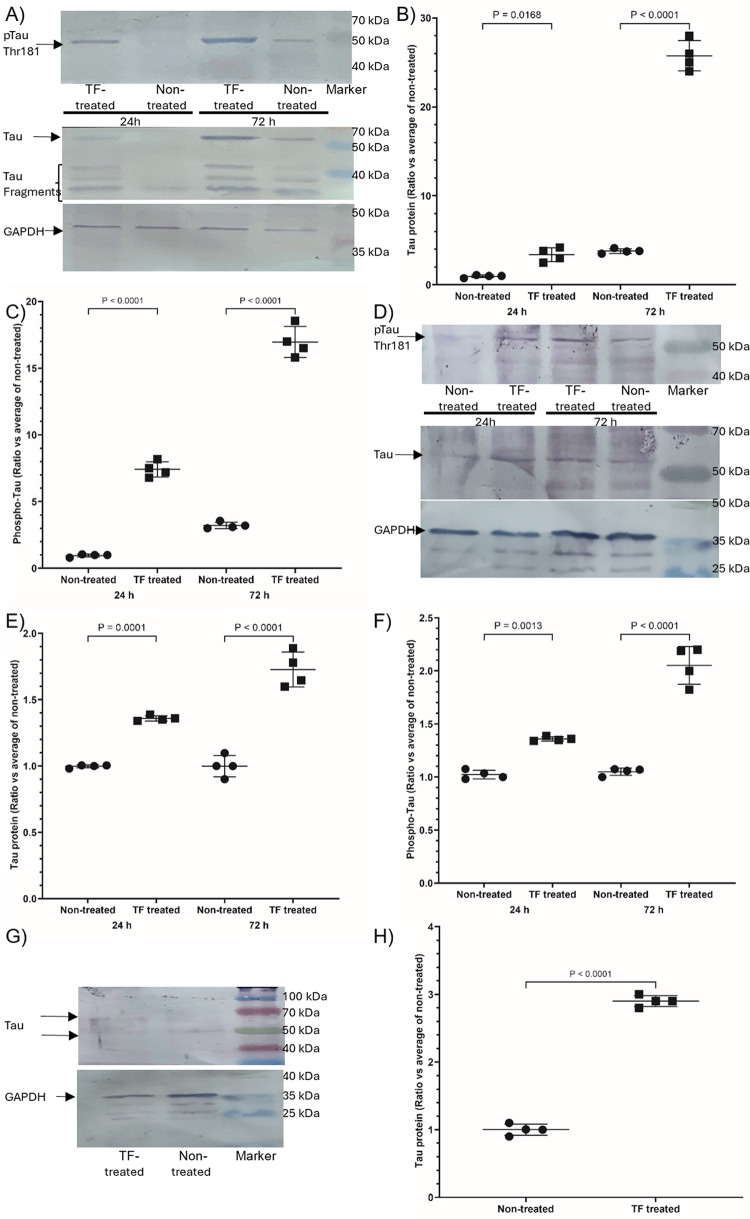
Analysis of the influence of TF on the expression and phosphorylation of Tau protein in differentiated SH-SY5Y, HCN-2, and rat neuronal cells. SH-SY5Y, HCN-2 and rat neuronal cells (2 × 10^5^) were treated with recombinant relipidated Innovin TF (0.65 ng/ml) and sets of the cells were harvested at 24 h. Another set of SH-SY5Y and HCN-2 cells were supplemented with a second dose at 48 h and harvested at 72 h. Cellular lysates (10 µg protein) were examined for Tau and phospho-Thr181 Tau by western blot analysis, and band densities were determined. All values were normalised against the respective GAPDH and for comparison, all ratios were calculated against the average from the non-treated cells at 24 h ± the calculated standard deviation. The data were obtained from 4 biological experiments, and all data groups were determined to have normal distributions. **A** SH-SY5Y electrophoresis, **B** SH-SY5Y Tau protein ratio, **C** SH-SY5Y phospho-Thr181 Tau ratio, **D** HCN-2 electrophoresis, **E** HCN-2 Tau protein ratio, **F** HCN-2 phospho-Thr181 Tau ratio, **G** rat neuron electrophoresis, **H** rat neuron Tau protein ratio (rat neuron phospho-Thr181 Tau was not analysed)

To assess the influence of TF in the absence of other EV proteins, SH-SY5Y, HCN-2 and rat neuronal cells were treated with recombinant TF (0.65 ng/ml, reconstituted in vesicles). Short term treatment of the cells (24 h) upregulated the expression of Tau protein reaching 4-, 1.4- and 3-fold in SH-SY5Y (Fig. 4A and B), HCN-2 (Fig. 4D and E) and rat neuronal cells (Fig. 4G and H), respectively. Repeated supplementation of the cells for 72 h, with recombinant TF enhanced the expression of Tau in SH-SY5Y (6.8-fold; Fig. 4A and B) and maintained the expression in HCN-2 cells (1.7-fold; Fig. 4D and E) but was not tested in rat neurons. In the absence of repeated supplementation with TF, the levels of Tau protein returned to those of the non-treated samples by 72 h (not shown).

### Exposure of Cells to TF Protein Alone Upregulates Tau Expression

**Fig. 5:**
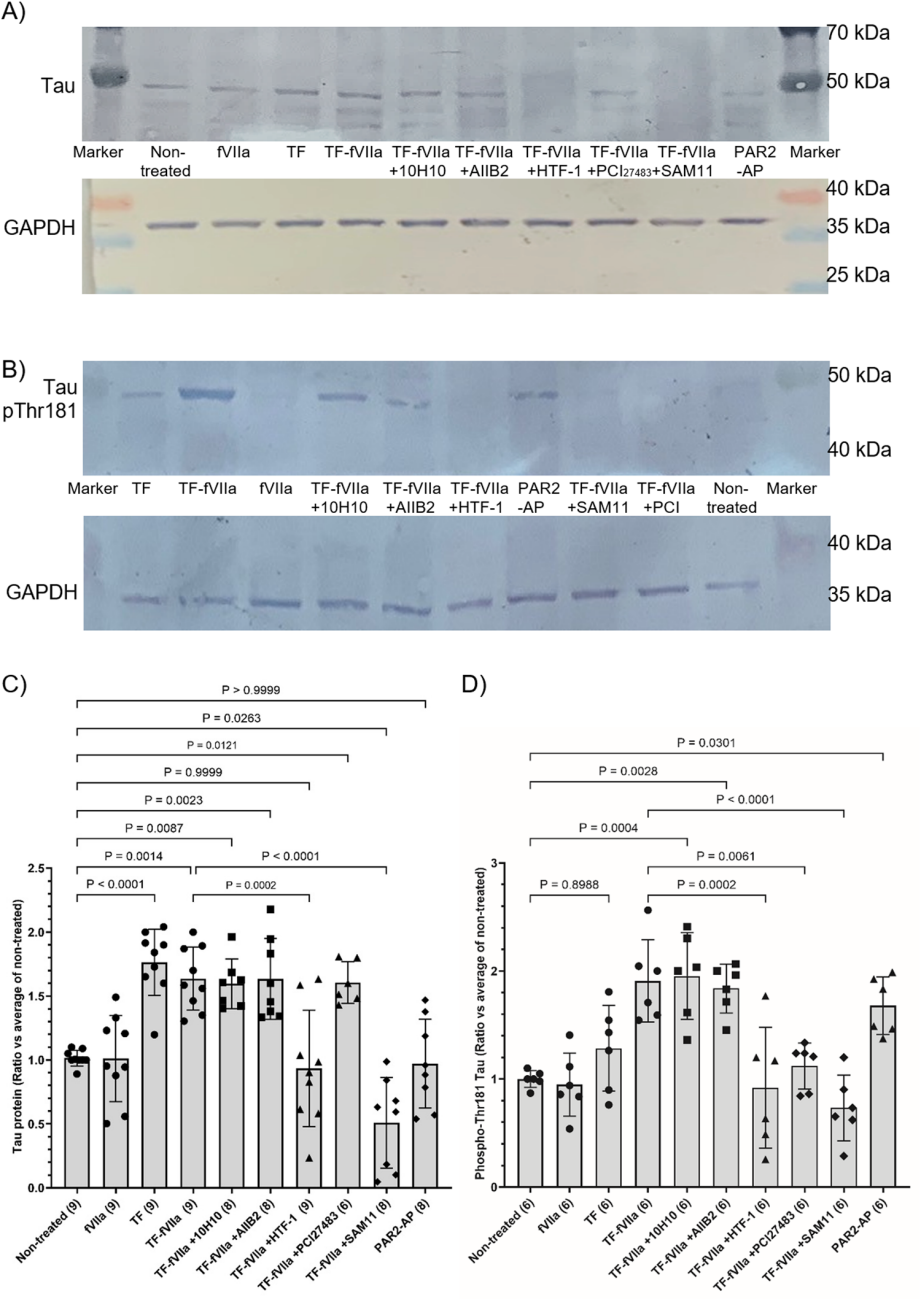
Examination of the mechanism of influence of TF on Tau protein expression and phosphorylation in differentiated SH-SY5Y cells. SH-SY5Y (2 × 10^5^) were treated with recombinant relipidated Innovin TF (0.65 ng/ml) together with or without human fVIIa (5 nM) or fVIIa alone. In some experiments, the TF aliquots were pre-incubated for 1 h, with 10H10 antibody (20 µg/ml) capable of blocking TF signalling, HTF-1 antibody (20 µg/ml) to block TF-fVIIa protease/procoagulant activity, or a mouse control isotype IgG antibody (20 µg/ml; not shown). In other experiments, fVIIa was pre-incubated for 1 h with the chemical inhibitor PCI27483 (10 µg/ml). Alternatively, the neuronal cells were treated with AIIB2 antibody (20 µg/ml) to block β1-integrin signalling, SAM11 antibody (20 µg/ml) capable of blocking PAR2 signalling, or PAR2-activating peptide (PAR2-AP; 20 µM) to induce PAR2 signalling. Cells were harvested at 24 h, and cellular lysates (10 µg protein) were examined for Tau and phospho-Thr181 Tau by western blot analysis. All values were normalised against the respective GAPDH and for comparison, all ratios were calculated against the average from the non-treated cells ± the calculated standard deviation. The number of experiments is shown in each column, and all data groups were determined to have normal distributions and are shown in each column. **A** SH-SY5Y protein electrophoresis, **B** SH-SY5Y phospho-Thr181 Tau electrophoresis, **C** SH-SY5Y Tau protein ratio, and **D** SH-SY5Y phospho-Thr181 Tau ratio

In order to determine the nature of the interaction of TF with the cells, and the underlying mechanism by which TF induces Tau expression, further examinations were carried out using SH-SY5Y cells. Measurements of the Tau mRNA and protein expression indicated that the mechanism of induction was not significantly altered by the presence of fVIIa (Figs. 3D and E and 5A and C), but in the absence of TF, supplementation with fVIIa did not have any influence (Fig. 5C). However, pre-incubation of TF with HTF-1 antibody to block the interaction of TF with fVIIa, or pre-incubation of the cells with SAM11 antibody to prevent PAR2 activation suppressed the upregulation of Tau. Moreover, independent activation of PAR2 using a PAR2-activating peptide (SLIGKV) did not have any influence on Tau expression. In the absence of recombinant TF, neither HTF-1 nor SAM11 had any significant influence on Tau expression (not shown). Western blot examination of SH-SY5Y cells indicated the presence of endogenous fVII, both as the zymogen and the activated form (Supplementary Fig. 3) while the amounts of fVII in HCN-2 cells, was barely detectable. Inhibition of TF-signalling using the 10H10 antibody, or by inhibiting β1-integein signalling with AIIB2 antibody did not interfere with the upregulation of Tau expression by TF.

### Treatment of Cells with TF-fVIIa Induces the Phosphorylation of Tau Through a Mechanism Requiring PAR2

**Fig. 6:**
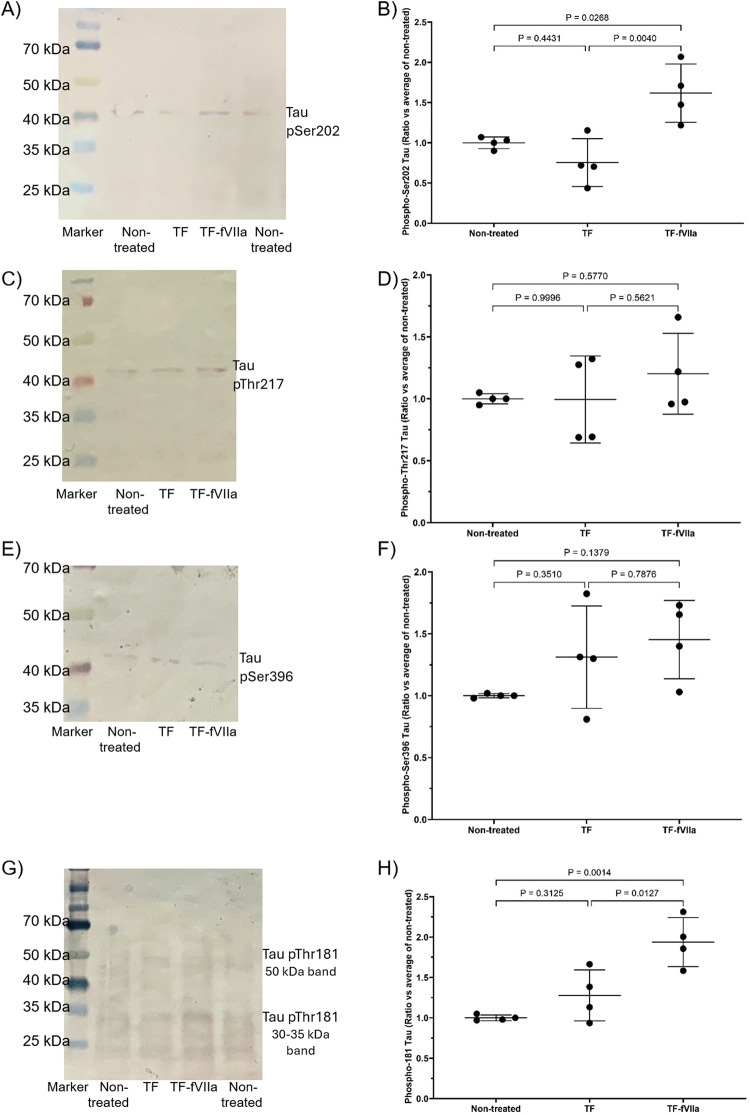
Examination of influence of TF on Tau phosphorylation at Ser202, Thr217, Ser396 and Thr181 in differentiated SH-SY5Y cells*.* SH-SY5Y (2 × 10^5^) were treated with recombinant relipidated Innovin TF (0.65 ng/ml) in the presence or absence of fVIIa (5 nM). Cells were harvested at 24 h, and cellular lysates (10 µg protein) were examined with **A** & **B** rabbit anti-human phospho-Ser202 Tau antibody, **C** & **D** rabbit anti-human phospho-Thr217 Tau antibody, **E** & **F** rabbit anti-human phospho-Ser396 Tau antibody, or **G** & **H** rabbit anti-human phospho-Thr181 Tau antibody. All values were normalised against the respective GAPDH (see Supplementary Fig. 5E) and for comparison, all ratios were calculated against the average from the non-treated cells ± the calculated standard deviation. The data were obtained from 4 biological experiments, and all data groups were determined to have normal distributions

**Fig. 7:**
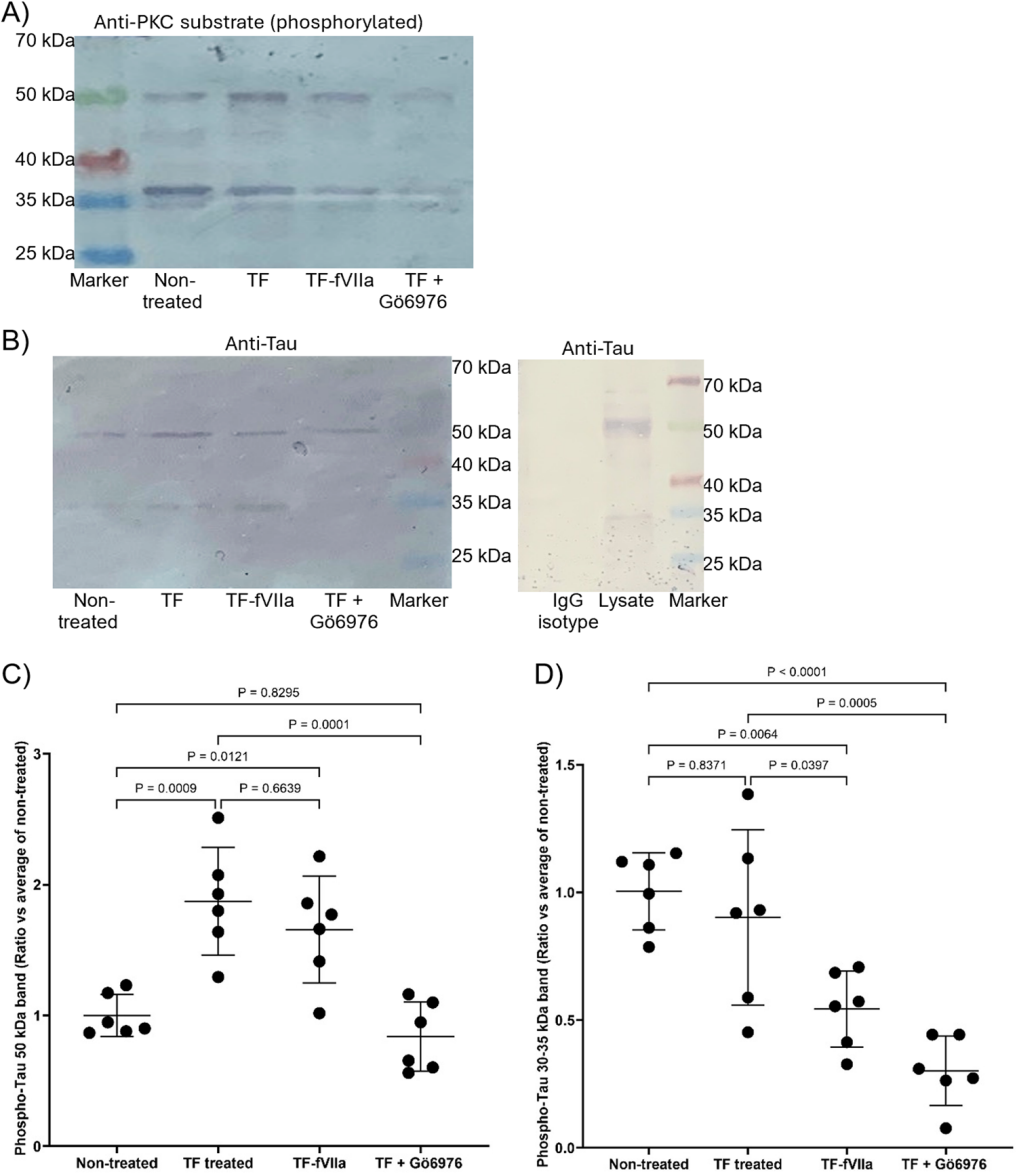
Examination of influence of TF on PKC-mediated Tau phosphorylation. SH-SY5Y cells (2 × 10^5^) were incubated with TF (0.65 ng/ml) in the presence or absence of fVII (5 nM) for 24 h. Additionally, to block the phosphorylation of Tau by PKC, cells were pre-incubated with the PKC inhibitor Gö6976 (100 nM) for 40 min, prior to addition of TF. Cells were then lysed in 500 µl of Phosphosafe lysis buffer containing a protease inhibitor cocktail (1% v/v). The cell lysates was incubated overnight with a mouse anti-human Tau antibody (2 µg per sample) alongside an IgG isotype control sample and immunoprecipitated using Pureproteome protein A-magnetic beads (10 µl). Cellular lysates (10 µg protein) were examined by western blot using (**A**) a rabbit anti-phospho-PKC-substrate motif antibody or (**B**) a rabbit anti-human Tau antibody. The bands were normalised against the immunoprecipitated tau bands. For comparison, the ratios of phosphorylated (PKC-substrate) bands at (**C**) 50 kDa and (**D**) 30–35 kDa were calculated against the average from the non-treated cells ± the calculated standard deviation. The data were obtained from 6 biological experiments. All data groups were determined to have normal distributions

Treatment of the cells with recombinant TF alone resulted in proportionally higher levels of Tau phosphorylation at Thr181, in SH-SY5Y cells than HCN-2 cells at 72 h (Fig. 4A, C, D and F) and was in line with the levels of endogenous fVII/fVIIa detected in these cells (Supplementary Fig. 3). The ratios of phospho-Thr181 Tau : total Tau protein were also calculated for SH-SY5Y and HCN-2 cells (Supplementary Fig. 4 A and 4B respectively). However, measurements of the induction of Thr181 Tau phosphorylation were concurrent with the upregulation of Tau expression, and the ratios of phospho-Thr181 Tau : Total Tau may vary depending on the time of sampling. Furthermore, incubation of SH-SY5Y cells with TF-fVIIa resulted in significant increases in the phosphorylation of Tau at Thr181 (Fig. 5B and D) but not on incubation with fVIIa alone. In support of these data, inhibition of protease activity of TF-fVIIa, either using the fVIIa inhibitor PCI27483, or by pre-incubation of TF with HTF-1 antibody suppressed the induction of Tau Thr181 phosphorylation. Furthermore, the phosphorylation of Thr181 was dependent on the activation of PAR2 alone, and pre-incubation of the cells with SAM11 antibody to prevent PAR2 activation completely suppressed Tau phosphorylation. However, again the calculated ratios for phospho-Thr181 Tau : Total Tau protein (Supplementary Fig. 4 C) were determined to be unrepresentative of the underlying mechanisms (see Discission). Examination of the phosphorylation of Ser202 in SH-SY5Y cells also indicated a small but significant increase following incubation with TF-fVIIa (Figs. 6A and B and S5A), while Thr217 (Figs. 6C and D and S5B) and Ser396 (Figs. 6E and F and S5C) remained unaffected. Immunoprecipitation of Tau protein followed by analysis with an antibody specific for phospho-PKC-substrate motif, indicated increased PKC-dependent phosphorylation of Tau presenting a band at around 50 kDa, following incubation of cells with TF alone. This phosphorylation was marginally lower on supplementation of TF with fVIIa and was preventable by pre-incubating the cells with PKC inhibitor Gö6976 (Fig. 7A, B and C). Additionally, a smaller Tau band (30–35 kDa) was detected and the analysis of the band densities indicated lower levels of PKC-dependent phosphorylation following treatment with TF-VIIa, compared to TF alone, or to the untreated sample (Fig. 7A, B and D).

### Prolonged Treatment of Cells with TF Promotes the Digestion of Tau

**Fig. 8:**
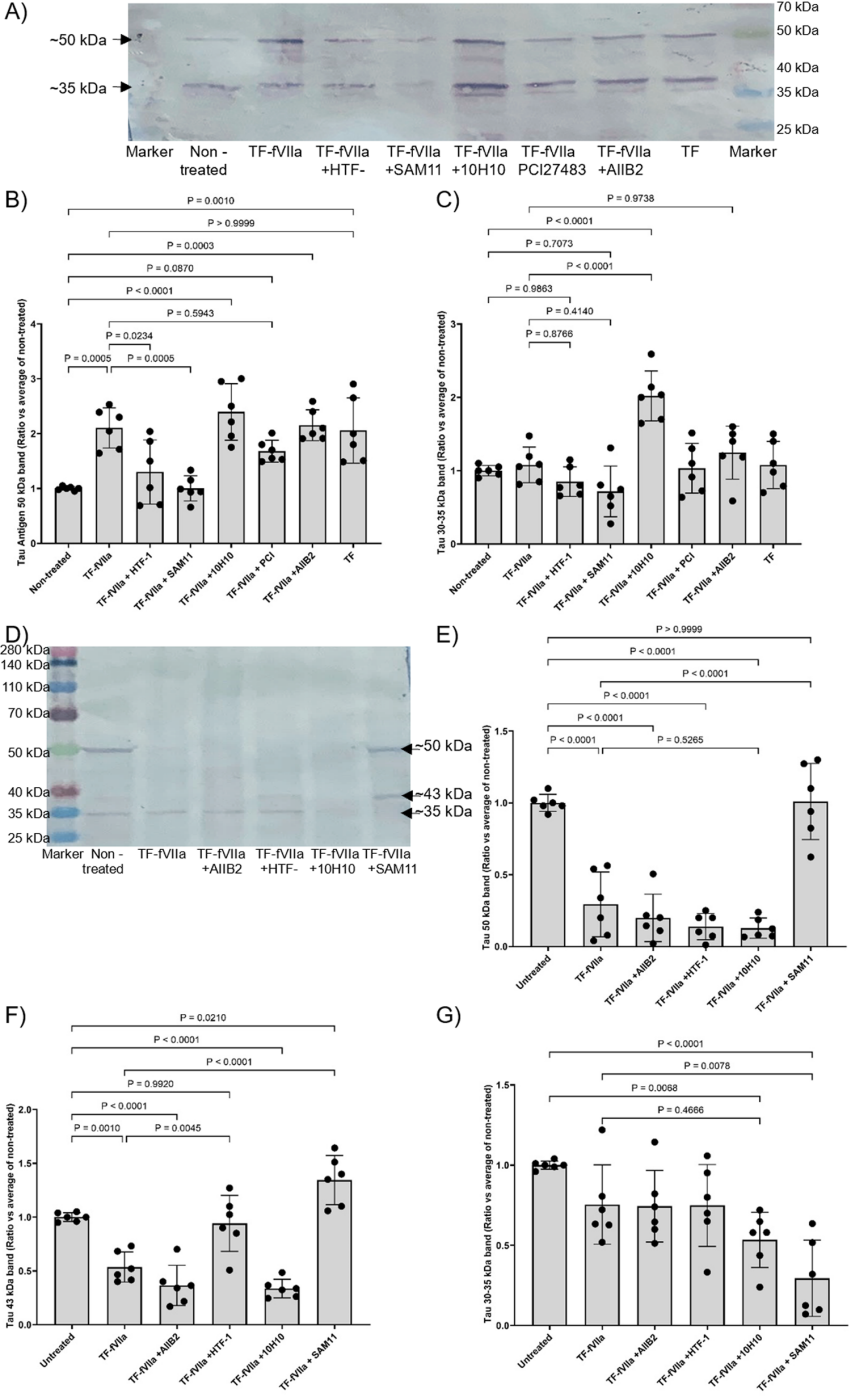
Time-course analysis of the Tau protein fragments in differentiated SH-SY5Y cells, following treatment with TF*.* SH-SY5Y (2 × 10^5^) were treated with as single dose of recombinant relipidated Innovin TF (0.65 ng/ml) together with human fVIIa (5 nM). In some experiments, the TF aliquots were pre-incubated for 1 h, with 10H10 antibody (20 µg/ml) capable of blocking TF signalling, HTF-1 antibody (20 µg/ml) to block TF-fVIIa protease/procoagulant activity, or a mouse control isotype IgG antibody (20 µg/ml; not shown). Alternatively, the neuronal cells were treated with AIIB2 antibody (20 µg/ml) to block β1-integrin signalling, or SAM11 antibody (20 µg/ml) capable of blocking PAR2 signalling. Sets of cells were harvested at **A** 48 h and **D** at 72 h and cellular lysates (10 µg protein) were examined for Tau by western blot analysis. All values were normalised against the respective GAPDH (see Supplementary Fig. 6 A and B) and for comparison, all ratios were calculated against the average from the non-treated cells ± the calculated standard deviation. The data were obtained from 6 biological experiments, and all data groups were determined to have normal distributions. **A** Electrophoresis at 48 h, **B** calculated ratios of 50 kDa bands at 48 h, **C** calculated ratios of 30–35 kDa bands at 48 h, **D** electrophoresis at 72 h, **E** calculated ratios of 50 kDa bands at 72 h, **F** calculated ratios of 40 kDa bands at 72 h, **G** calculated ratios of 30–35 kDa bands at 72 h

To further explore the outcome of Tau phosphorylation in response to TF-fVIIa, the sizes and distributions of the three major Tau bands, observed in SH-SY5Y cells were further examined at 48 and 72 h, post-treatment and normalised against the respective GAPDH. Assessment of the bands at ~ 50 kDa indicated an initial increase in this band on all treatments except in the presence of HTF-1 and SAM11 antibodies (Fig. 8A and B). This rise was followed by a decline by 72 h, but inhibition of PAR2 using SAM11 antibody prevented the decline in this band, at 72 h (Fig. 8D and E). The levels of GAPDH remained unaltered at these time points (Supplementary Fig. 6 A and B respectively) and the ratios of the 50:30 kDa bands (Supplementary Fig. 6 C and E) and the 50:40 kDa bands were also calculated (Supplementary Fig. 6D). This band coincided with the Thr181-phosphorylated band (Figs. 5B and 6G). A separate 43 kDa band was detectable at 72 h, in the untreated sample, and in the sample where PAR2 was inhibited (Fig. 8D and F). This band was also present to lesser amount in the sample where TF-fVIIa activity was inhibited using HTF-1 antibody (Fig. 8D and F). Interestingly, this band coincided with the molecular weight of the Ser202, Thr217 and Ser396 phosphorylated bands (Fig. 6A, C and E). Finally, treatment of cells with TF-fVIIa did not cause a significant change in the ~ 30–35 kDa band at 48 h (Fig. 8A and C), which was shown to be phosphorylated at Thr181 (Fig. 6G). This band was marginally reduced at 72 h but significantly reduced by PAR2 inhibition (Fig. 8D and G).

### Prolonged Treatment of Cells with TF Promotes the Accumulation of Tau Fragments and the Formation of Aggregates

**Fig. 9:**
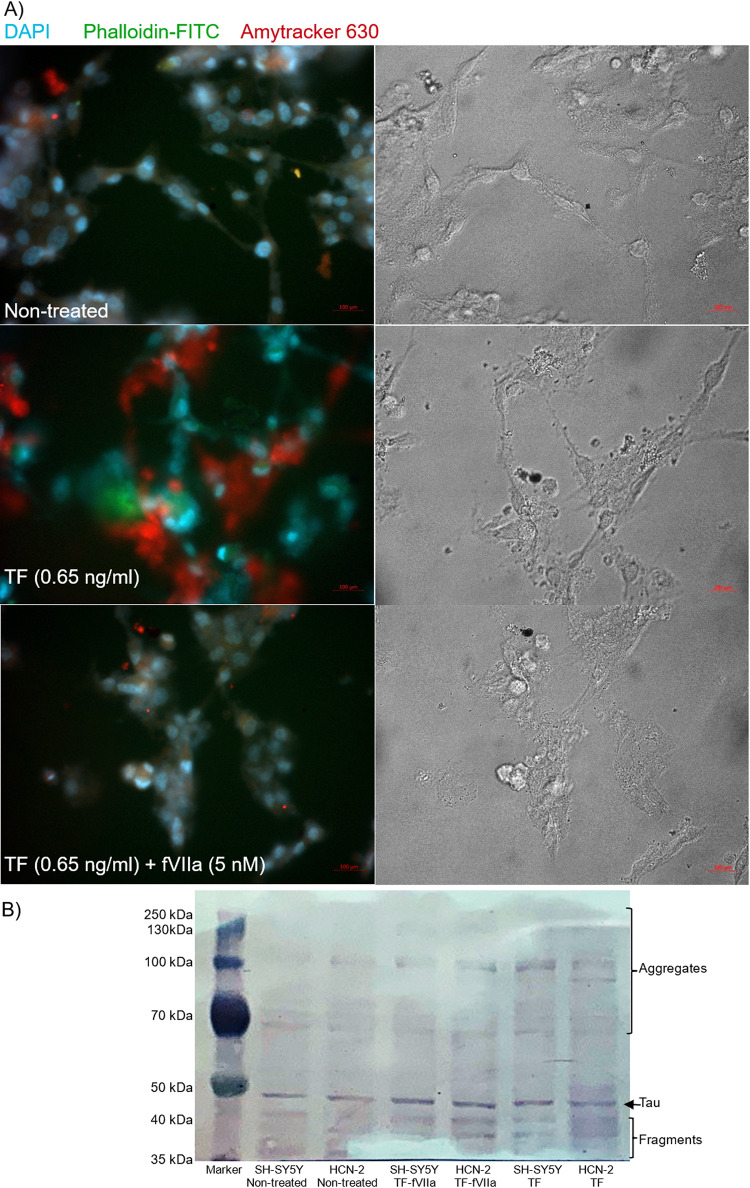
Examination of the formation of aggregates in response to TF. **A** SH-SY5Y were plated in 29 mm culture dishes with a 10 mm glass bottomed micro-well and differentiated as above. Aliquots of the differentiated cells were treated daily with TF (0.65 ng/ml) in the presence and absence of fVIIa (5 nM) for up to 3 days. The cells were then fixed, washed and probed with Amytracker 630 (1 µg/ml) in distilled water. The cells were stained with DAPI (2 µg/ml) and Phalloidin-iFluor 488 (2 µg/ml) and images were acquired at room temperature, using a Zeiss Axio Vert.A1 inverted fluorescence microscope at × 40 magnification. Images were acquired using the ZEN software and the filters were selected for DAPI, FITC and Texas Red. (Images are representative of 3 separate experiments). **B** SH-SY5Y cells (2 × 10^5^) were repeatedly treated at the start and at 48 h, with recombinant relipidated Innovin TF (0.65 ng/ml) in the presence and absence of human fVIIa (5 nM). The conditioned media were collected at 72 h and centrifuged at 3000 *g* for 5 mins and the proteins were then concentrated using a Centricon concentrators with 3 kDa cutoff and centrifuged at 3,000 *g* for 5 h at 4 °C. The retained proteins were then examined for Tau by western blot analysis. (Images are representative of 3 separate experiments)

Treatment of SH-SY5Y cells with recombinant TF alone resulted in formation of aggregates within 72 h, as indicated by the uptake of the Amytracker 630 stain (Figs. 9A and S7). In comparison, inclusion of fVIIa together with recombinant TF noticeably reduced, but did not eliminate the size or the intensity of the aggregates. Qualitative examination of conditioned media from SH-SY5Y and HCN-2 cells indicated the presence of Tau aggregates of > 70 kDa, as well as the presence of fragments (Fig. 9B). Furthermore, the majority of the detectable fragments appeared to be phosphorylated at Thr181 (Supplementary Fig. 8). The measured protein concentrations did not significantly vary between the sets of conditioned media from the treated and untreated samples, for either cell type, which were concentrated using Amicon filters.

### Examination of the Digestion of Tau by TF-fVIIa Protease

**Fig. 10:**
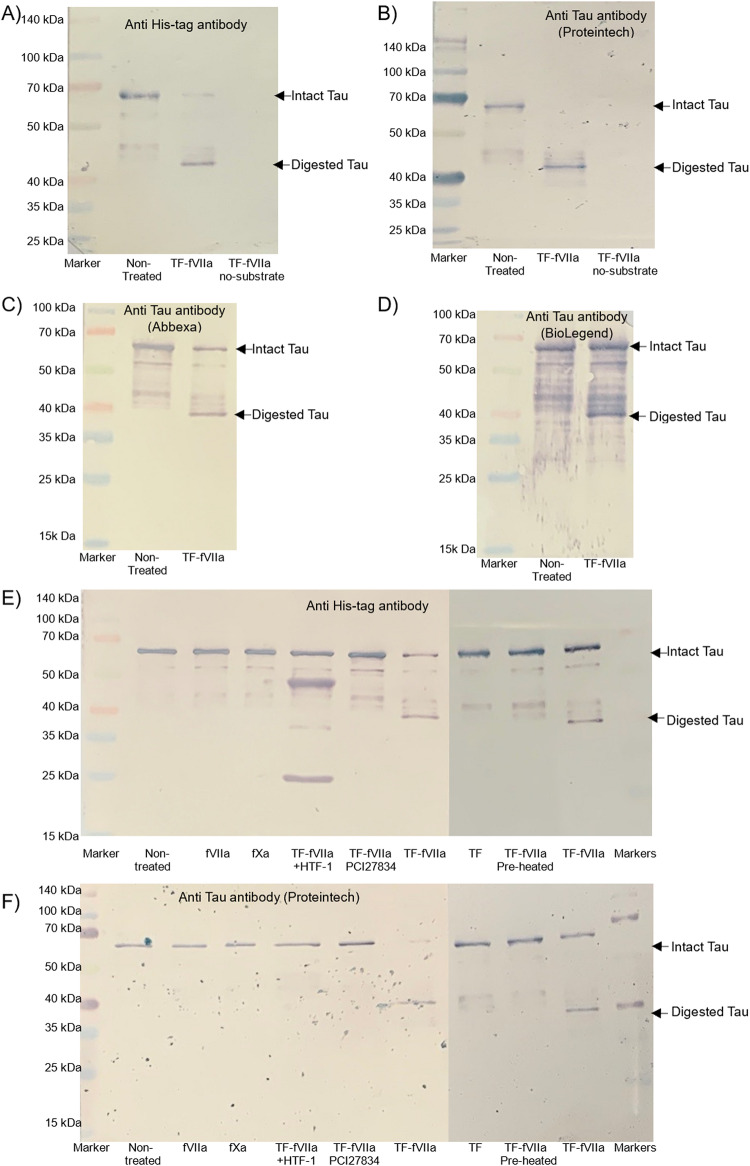
Analysis of digestion of recombinant Tau protein by TF-fVIIa. Recombinant full-length Tau-His tag (Tau-441; 40 µg/ml) was digested with TF-fVIIa complex. Reactions were prepared to include recombinant TF (1.3 ng/ml) and fVIIa (10 nM), Tris-HCl pH 7.4 (5 mM) and CaCl_2_ (5 mM) and incubated for 1 h at 37 °C. Sets of the proteins were then examined by western blot, probed with **A** an alkaline phosphates-conjugated anti-His Tag antibody, and rabbit anti-Tau antibodies from **B** Proteintech, **C** Abbexa and **D** mouse anti-Tau antibody from BioLegend. (Images are representative of 3 separate experiments). In some reactions, the recombinant TF was pre-incubated with HTF-1 antibody (20 µg/ml) to inhibit the protease function of TF-fVIIa, or supplemented with fVIIa inhibitor, PCI27483 (10 µg/ml). Separate samples of the recombinant Tau were incubated with fVIIa (10 nM) alone, or fXa (10 nM) or recombinant TF (1.3 ng/ml). Additionally, samples of TF-fVIIa were preheated at 95 °C for 10 min prior to addition to recombinant Tau. The samples were then examined western blot and probed using **E** an alkaline phosphates-conjugated anti-His Tag antibody, and **F** a rabbit anti-Tau antibody from Proteintech, and developed and visualised as above. (Images are representative of 3 separate experiments)

In order to examine the possibility that TF-fVIIa may be capable of digesting the Tau, recombinant full-length His tag-Tau protein (with the tag on the N-terminal) was incubated with recombinant TF and fVIIa. The fragments were then analysed by western blot and detected using HRP-conjugated anti-His tag (Fig. 10A), as well as using two separate polyclonal rabbit anti-human Tau antibodies (Fig. 10B and C), and also using a monoclonal mouse anti-human Tau antibody (Fig. 10D). Digestion of the protein with TF-fVIIa reduced the band intensity of the intact recombinant Tau (Mw 67 kDa including the tag) and resulted in the appearance of a band at 40 kDa which was detectable using the anti-His tag antibody, or either of the anti-Tau antibodies. Moreover, pre-incubation of the TF with an inhibitory antibody (HTF-1), or pre-incubation of TF-fVIIa with the inhibitor PCI27483 partially prevented the appearance of the 40 kDa band (Fig. 10E and F). However, in the presence of HTF-1 the light and heavy chains for the HTF-1 antibody were also labelled and visible. Incubation of the recombinant His tag-Tau protein with either TF, fVIIa individually or fXa alone did not result in the appearance of the 40 kDa. Finally, pre-incubation of TF-fVIIa at 95 °C for 10 min prior to addition of the His tag-Tau protein largely prevented the digestion of Tau.

## Discussion

Neurodegenerative conditions are characterized by the progressive deterioration of the brain and/or the peripheral nervous system and remain incurable. The relationship between inflammation and neuropathies (diseases of brain and nerves) is well known. Some inflammation is essential for the repair of the injured nerves, but prolonged inflammation causes impairment of nerve cell function, as well as the deposition of plaques within the brain which cause many neuropathies. Tissue factor (TF) is a protein that may link inflammation with the onset and progress of neuropathies. The brain is particularly rich in TF which rapidly initiates blood clotting to prevent bleeding, since any bleeding within the brain can be fatal. TF also induces cellular signalling that prompts the clear-up of damaged cells and initiates repair processes. Therefore, TF may induce both protective and detrimental effects and excessive amounts are known to collect around the plaques within the brain of patients with Alzheimer’s, and released in cerebrospinal fluid of Parkinson’s patients, but not in healthy brains (Leung et al. 2015; McComb et al. 1991). However, any direct influences arising from TF-associated signalling on neuronal cells have not been determined.

Our data indicate that stimulation of neuronal cells with TF induced the upregulation of Tau protein and mRNA in the three neuronal cells tested. Tau upregulation was more pronounced in SH-SY5Y cells following incubation with recombinant TF since these cells appear to express endogenous fVIIa. Blocking of the interaction of TF with fVIIa, using HTF-1 antibody neutralised Tau upregulation while prevention of PAR2 activation using SAM11 antibody suppressed Tau expression. However, additional supplementation with fVIIa did not enhance Tau expression and inclusion of the fVIIa inhibitor PCI27483 did not prevent the upregulation. Due to the small number of available cells, in the current study, it was not possible to carry out all the experiments using HCN-2 cells, in order to determine the influence and outcome of various antibodies. However, our data indicate that the interaction of TF with the cells is sufficient to induce Tau expression, and the contribution of PAR2 to this mechanism appears to be as a trigger, similar to our previously reported observations in other cells (Ettelaie et al. 2022; Featherby and Ettelaie 2024). Moreover, to examine this hypothesis, it is essential to determine the influence of a range of concentrations of TF, in conjunction with fVIIa and/or PAR2 blockers.

The upregulation of Tau was concurrent with increased phosphorylation of Tau protein and was mediated through PAR2 signalling. However, expression of Tau, its post-translational modifications, fragmentation, and release from cells occur as discrete events and can influence the measurements. The findings from our study, illustrating the outcome of treatments on Tau-mediated processes are summarised in Table 1. We propose that the expression of Tau is induced by TF alone but requiring the activation of PAR2 which acts as an initiating trigger. In contrast, induction of Tau phosphorylation requires TF-fVIIa protease activity and is dependent on PAR2 activation. The phosphorylation of Tau appears to be associated with the degradation and release of Tau (Hanger et al. 2009; Sattarov et al. 2024; Wegmann et al. 2021) which further alters the levels of Tau bands that are detected at various time-points. Therefore, the calculated ratio of phospho-Tau to Tau protein is a product of dynamically regulated Tau turnover. In order to determine the precise mechanism of the phosphorylation of Tau following the exposure of neurons to TF-EV, it is essential to (a) identify the kinases responsible for the phosphorylation of various specific residues and, (b) to examine the phosphorylation of these residues at shorter periods following exposure to TF-EV, so as to represent kinase activation.

**Table 1:** Summary of the outcome of the treatments on Tau expression, phosphorylation, degradation/digestion, aggregation and release

Influence on Tau	EV	TF	TF-fVIIa	TF-fVIIa	TF-fVIIa	PAR2
				+HTF-1	+SAM11	-AP
Tau mRNA expression	**↑**	↑	↑			
Tau protein expression	↑	↑	↑	N/C	↓	N/C
Tau phosphorylation Thr181	↑	_ꜛ_	↑	N/C	↓	↑
Tau phosphorylation Ser202		N/C	↑			
Tau phosphorylation Thr217		N/C	N/C			
Tau phosphorylation Ser396		N/C	_ꜛ_			
Tau phosphorylation (PKC)		↑	↑			
Tau release from cells		↑	↑			
Tau aggregation		↑	_ꜛ_			
30 kDa fragment appearance		N/C	N/C	N/C	↓	
40 kDa fragment appearance		N/C	N/C	↑	↑	
Recombinant Tau digestion		N/C	↑	N/C		

↑ = Increase; ꜛ = Marginal/non-significant increase; N/C = No significant change; ↓ = Decrease; Empty spaces = not tested

As stated above, phosphorylation of Thr181 was significantly enhanced on treatment with the combination of TF-fVIIa, compared to TF or fVIIa individually and required the proteolytic activity of fVIIa. Phosphorylated Tau bands of around 50 and 30–35 kDa were detected at 24 h, but then subsided below that of the untreated sample, by 72 h (Fig. 4). Phosphorylation of Thr181 has been associated with the release of an N-terminal fragment of Tau into the cerebrospinal fluid (Wegmann et al. 2021). Moreover, increased Thr181-phosphorylation, but not Thr217-phosphorylation of Tau is associated with the incorporation and release of Tau within cell-derived extracellular vesicles (EV) in both Alzheimer’s patients and normal individuals (Sattarov et al. 2024). These EV were reported to be of up to 300 nm in diameter and therefore, similar in size to the procoagulant EV released following the activation of PAR2 by TF-fVIIa (Ettelaie et al. 2014). Moreover, phosphorylation of Thr181 has been suggested to represent axonal alteration/abnormality (Hirota et al. 2022) but little difference within cerebrospinal fluid was reported between Alzheimer’s and control individuals (Meredith et al. 2013). Considering the role of Thr181 in co-ordinating Tau hyperphosphorylation (Stefanoska et al. 2022) this modification may represent a physiological response and may not necessarily be disease-associated (Wegmann et al. 2021). High levels of cellular TF can activate p38α (ElKeeb et al. 2015; Ethaeb et al. 2020) and Src-1 (Ethaeb et al. 2020) and these kinases are reported to promote the hyperphosphorylation of Tau protein (Nelson et al. 2009; Stefanoska et al. 2022). Abnormal hyperphosphorylation of Tau has in turn, been suggested to promote Tau proteolysis and aggregation (Hanger et al. 2009). However, neither phosphorylation nor proteolytic digestion of Tau is toxic to the cells (Wang et al. 2007). Furthermore, specific phosphorylation patterns are known to prevent proteolysis by specific enzymes (Arai et al. 2005). Notably, the phosphorylation of Thr181 on Tau protein can be mediated by p38α kinase (Maphis et al. 2016; Stefanoska et al. 2022), and results in the two-phased elevations of Tau release into the serum (Randall et al. 2013) but may also contribute to the formation of paired-helical filaments (Holper et al. 2022). Phospho-Tau has been shown to localise to plasma membrane which may then be released within cell-surface derived extracellular vesicles (Dujardin et al. 2014; Georgieva et al. 2014; Klein et al. 2002). Since activation of PAR2 results in rapid incorporation and release of TF-fVIIa complex within EV, these vesicles may also harbour Tau protein or fragments derived from it. The digestion of Tau by soluble proteases such as thrombin has been demonstrated (Arai et al. 2005). In our study, digestion of recombinant His tag-Tau by TF-fVIIa but not fXa, released a peptide of the 40 kDa (including the N-terminal tag). An examination of Tau protein sequence indicated a likely cleavage site for fVIIa, at Arg_169_-Ile_170_ bond. In fact, the Asn-Ala-Thr-Arg_169_-Ile_170_ sequence closely resembles the fVIIa-substrate recognition sites on fXa (Asn-Val-Thr-Arg-Ile) and fIXa (Asn-Val-Ile-Arg-Ile). We previously demonstrated the use of the Asn-Val-Thr-Arg-Ile peptide as substrate for TF-fVIIa proteolysis (Featherby et al. 2019). Therefore, it is possible that the digestion of Tau at the cell membrane allows for incorporation and physiological release of Tau. Such a mechanism may constitute a means of cellular response to injury, to permit the clearance of damaged Tau from injured cells, without the formation of aggregates, while concurrently inducing the expression of new Tau mRNA.

However, this site is also part of the sequence that may be phosphorylated by protein kinase C α (PKCα) with 4th highest score in the Tau sequence, when analysed for PKC phosphorylation site prediction (Blom et al. 1999). Induction of PAR2 signalling by TF-fVIIa induces PKCα kinase activity (Ahamed and Ruf 2004). In turn, PKC isozymes are known to phosphorylate and alter the metabolism of Tau (Korulu et al. 2013; Leem et al. 2009; Taniguchi et al. 2001). Therefore, it is conceivable that the phosphorylation of threonine within this sequence may render the site inaccessible to TF-fVIIa digestion, and protects Tau from further degradation by TF-fVIIa. As these events may occur at different time-points, the activation of PAR2 may promote the delayed phosphorylation of this site by PKC, which in turn may act as a means of protecting Tau and preventing excessive degradation of Tau. Alternatively, SH-SY5Y cell-expressed fVIIa would interact with exogenous TF, localising at the surface of cells, and may be sufficient to digest Tau protein, but not to activate PAR2.

Incubation of cells with TF alone resulted in formation of the aggregates while inclusion of fVIIa with TF reduced aggregate formation and enhanced cell extensions. TF is a highly pro-inflammatory protein the retention of which results in over-activation of p38α kinase (ElKeeb et al. 2015). It is feasible that prolonged exposure of cells to high levels of TF as a result of inflammation, may promote dissimilar phosphorylation patterns by p38α kinase, which may then lead to unorthodox digestion of Tau and formation of the aggregate. Inhibition of PAR2 and to a lesser extend blocking of TF-fVIIa activity reduced the phosphorylation of the 50 kDa Tau band and resulted in the appearance of a different Tau band at around 43 kDa which was not phosphorylated at Thr181. This band was phosphorylated at the other residues tested (Ser202, Thr217 and Ser396) but only the level of phospho-Ser202 increased following TF-fVIIa treatment. Phosphorylation of Ser202 has been observed during chronic traumatic encephalopathy, following repetitive head impacts (Stathas et al. 2022) and may also occur by p38α kinase (Maphis et al. 2016). Such prolonged exposure to high levels of TF, in the absence of fVIIa may be promoted by inflammatory mediators released by various cells, in vivo. Consequently, prolonged levels of fVIIa-deficient TF-EV within the brain tissue may be one link between chronic inflammation and neuropathies.

The turnover and correct processing of microtubule proteins is essential during appropriate neuronal homeostasis and for normal function. However, dysregulations in these processes are associated with neuropathies. Our data demonstrate that TF signalling influences neuronal cells by increasing the expression of Tau. We hypothesise that upon injury, the signalling mediated through TF and PAR2, promotes the appropriate turnover of Tau protein and may encourage neuronal regeneration. However, chronic inflammatory conditions encourage prolonged exposure to TF in the absence of fVIIa, and promote inappropriate processing of Tau protein that is detrimental to the cells.

## Supplementary Information

Below is the link to the electronic supplementary material.

Supplementary material 1 (PDF 957.9 kb)

## Data Availability

All data generated or analysed during this study are included in this published article.
