# Supplementary material for: Procoagulant Extracellular Vesicles Increase Neuronal Tau expression, Metabolism and Processing Through Tissue Factor and Protease Activated Receptor 2
Source: Cell Mol Neurobiol. 2026 Jan 6;46:21. doi: 10.1007/s10571-025-01658-7 (PMC12847580; doi:10.1007/s10571-025-01658-7)
Supplement: Supplementary file 1 — Supplementary material 1 (PDF 957.9 kb) [file 10571_2025_1658_MOESM1_ESM.pdf]

Supplementary Figure 1: Cells lines

A) SH-SY5Y cell line was obtained from European Collection of Authenticated Cell Cultures

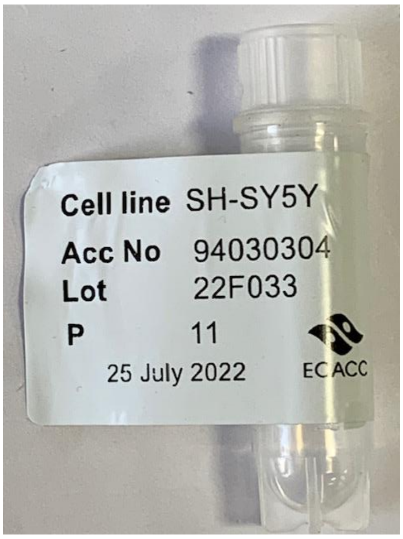

B) HCN-2 cell line was obtained from American Type Culture Collection

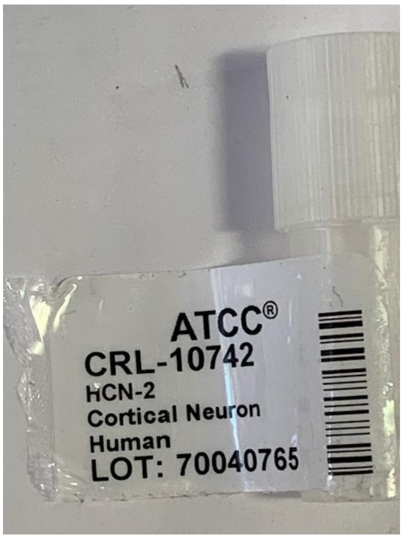

Supplementary Table 1: List of antibodies, the suppliers and catalogue numbers

Antibody	Provider	Catalogue No
Microtubule-Associated Protein Tau Antibody	Abbexa	abx012906
Microtubule-Associated Protein Tau Phospho-Thr181 (MAPT pT181) Antibody	Abbexa	abx119537
Microtubule Associated Protein Tau Phospho-Ser519/202 (MAPT pS519/202) Antibody	Abbexa	abx012588
Microtubule Associated Protein Tau Phospho-Thr534/217 (MAPT pT534/217) Antibody	Abbexa	abx012589
Microtubule-Associated Protein Tau Phospho-Ser396 (MAPT pS396) Antibody	Abbexa	abx012570
Doublecortin (DCX) Antibody	Abbexa	abx119035
Neuro D (Phospho-Ser274) Antibody	Abbexa	abx012701
Synaptophysin (SYP) Antibody	Abbexa	abx013199
Microtubule Associated Protein 2 (MAP2) Antibody	Abbexa	abx002009
Neurofilament, Light Polypeptide (NEFL) Antibody	Abbexa	abx312950
Tubulin Beta 3 (TUBB3) Antibody	Abbexa	abx110305
Gamma-Enolase (ENO2) Antibody	Abbexa	abx013157
Activity-Regulated Cytoskeleton-Associated Protein	Abbexa	abx123749
Ubiquitin Carboxyl Terminal Hydrolase L1 (UCHL1) Antibody	Abbexa	abx013178
Tau Polyclonal antibody	Proteintech	10274-1-AP
Phospho-Tau (Thr181) Polyclonal antibody	Proteintech	28866-1-AP
PSD-95 Antibody	Proteintech	81106-1-RR
HRP anti-Tau,210-230 Antibody	BioLegend	806405
Goat anti-rabbit IgG-AP Antibody	Santa Cruz	sc-2007
Goat anti-mouse IgG-AP Antibody	Santa Cruz	sc-2008
Donkey anti-goat IgG-AP Antibody	Santa Cruz	sc-2022
Goat anti-human GAPDH (V18) Antibody	Santa Cruz	sc-20357
Mouse anti-PAR2 (Clone SAM11) antibody	Santa Cruz	sc-13504

Mouse anti- His-Tag Antibody (Clone H-3) antibody	Santa Cruz	sc-8036
Mouse anti-human CD142 (Clone 10H10) antibody	Bio-Rad	9010-5059
Mouse anti-human CD142 (Clone HTF-1) antibody	eBiosicence	16-1429-85
Rat anti-human CD29 (Clone AIIB2) antibody	DSHB	AIIB2-c
Rabbit anti-P-(S) PKC substrate antibody	Cell Signalling	2261S
Rabbit IgG isotype control (Clone DA1E) antibody	Cell Signalling	3900S
Mouse IgG isotype control (Clone G3A1) antibody	Cell Signalling	5415S

Abbexa Ltd, Cambridge, UK

BioLegend, London, UK

Bio-Rad Laboratories, Inc., Hemel Hempstead, UK

Cell Signalling, Technology, Leiden, Netherlands

Development Studies Hybridoma Bank, Iowa City, USA

eBioscience/Thermo Fisher Scientific, Warrington, UK

Proteintech, Manchester, UK

Santa Cruz Biotechnology, Heidelberg, Germany

Supplementary Table 2: Average length of outgrowths in three cell samples

Cell	Untreated	TF-treated
SH-SY5Y	5.6 ± 26.7 μm	124.7 ± 148.2 μm
HCN-2	7.6 ± 20.9 μm	47.5 ± 59.1 μm
Rat neurons	93.8 ± 101.5 μm	174.9 ± 173.3 μm

SH-SY5Y, HCN-2 and rat neuronal cells were plated in 29 mm culture dishes with a 10 mm glass bottomed micro-well and differentiated. Aliquots of the differentiated cells were treated daily with TF (0.65 ng/ml) and fVIIa (5 nM) for up to 3 days. cellular outgrowth (indicated with arrows) and connectivity were monitored by light microscopy on a Nikon TMS microscope with a camera attachment. Size determination was performed using a stage micrometre. The length of the outgrowths was determined using ImageJ software. A mask was created in each image and the length of the continuous connections between points was then analysed. In total 5 images each, from 3 experiments were analysed.

Supplementary Figure 2: Analysis of markers of neurite growth and synaptic formation

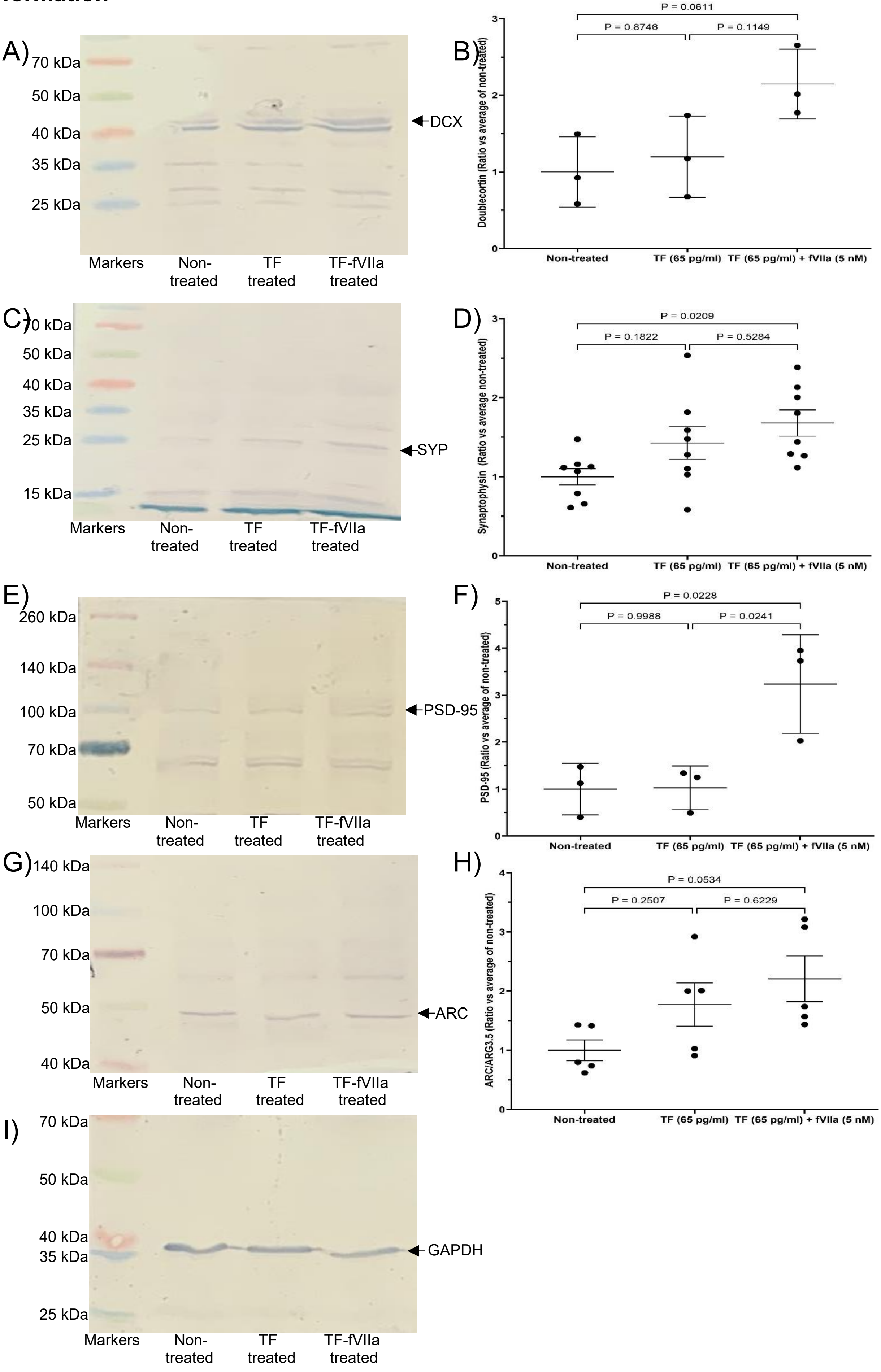

SH-SY5Y cells (2 × 10^5) were treated with recombinant relipidated Innovin TF (0.65 ng/ml) in the presence or absence of fVIIa (5 nM), harvested at 24 h and aliquots separated by denaturing 12% (w/v) polyacrylamide gel electrophoresis, transferred to a nitrocellulose membrane and blocked with TBST containing 1% (w/v) BSA. The membranes were separately probed overnight at 4˚C with rabbit anti-human Doublecortin, rabbit anti-human Synaptophysin, rabbit anti-human PSD-95, rabbit anti-human Activity-regulated cytoskeleton-associated protein (ARC) and goat anti-human GAPDH antibody, all diluted 1:3,000 (v/v) in TBST. The membranes were then developed with goat anti-rabbit IgG alkaline phosphatase-conjugated antibody or donkey anti-goat IgG alkaline phosphatase-conjugated antibody, diluted 1:3,000 (v/v) in TBST. The bands were visualised, and band densities were determined using the ImageJ software. All values were normalised against the respective GAPDH and for comparison, all ratios were calculated against the average from the non-treated cells ± the calculated standard deviation. The experiments were repeated 3 times for Doublecortin, 8 times for Synaptophysin, 3 times for PSD-95 and 5 times for Activity-regulated cytoskeleton-associated protein.

Supplementary Figure 3: Examination of the expression of TF and fVII protein in SH-SY5Y and HCN-2 cells

A) B)

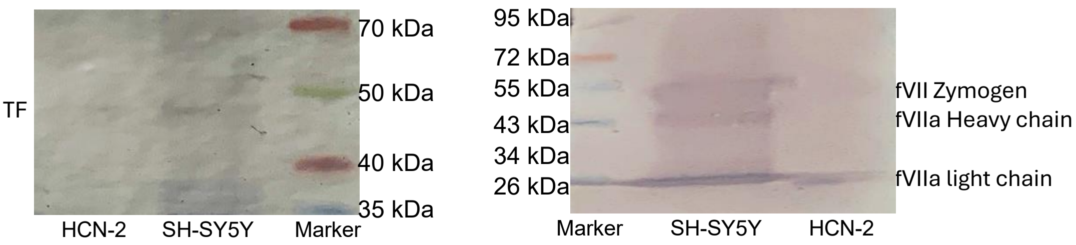

SH-SY5Y and HCN-2 cells (2×10^5) were lysed, protein content determined, and aliquots heated in Laemmli buffer. The lysates (10 µg protein) were separated by electrophoresis carried out on a denaturing 12% (w/v) polyacrylamide gel, transferred to a nitrocellulose membrane and blocked with TBST containing 1% (w/v) BSA. The membranes were probed overnight at 4˚C with A) a monoclonal antibody against TF (10H10) or B) a polyclonal rabbit anti-human factor VII antibody, diluted 1:3,000 (v/v) in TBST. Membranes were then washed and developed at room temperature for 60 min with goat anti-mouse IgG or anti-rabbit IgG alkaline phosphatase-conjugated antibody, diluted 1:3,000 (v/v) in TBST. The bands were visualised using the Western Blue stabilised alkaline phosphatase-substrate and recorded. (Images are representative of 3 separate experiments).

Supplementary Figure 4: Calculated ratios of phospho-Thr181 : Total Tau following treatment with TF and related reagents

A)

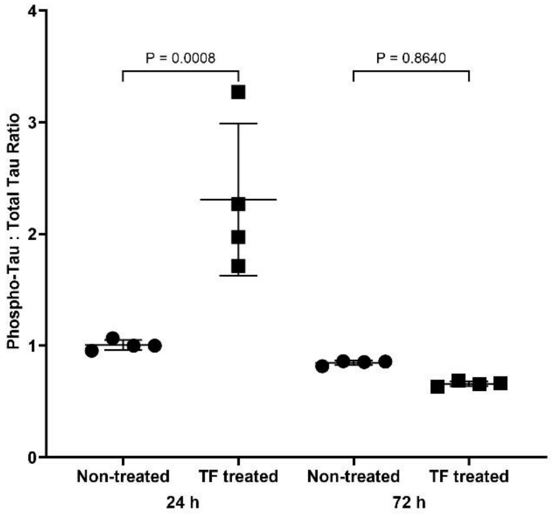

B)

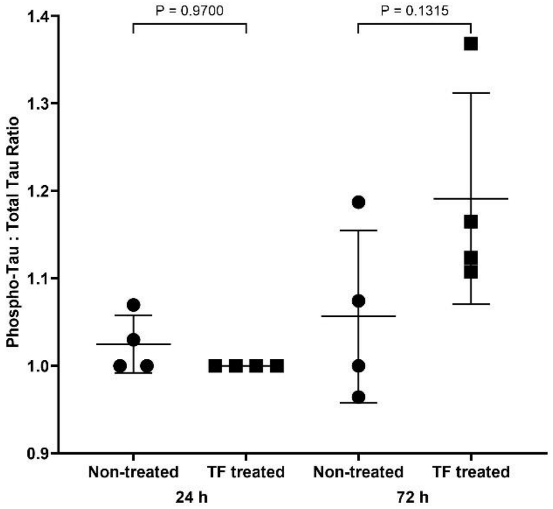

C)

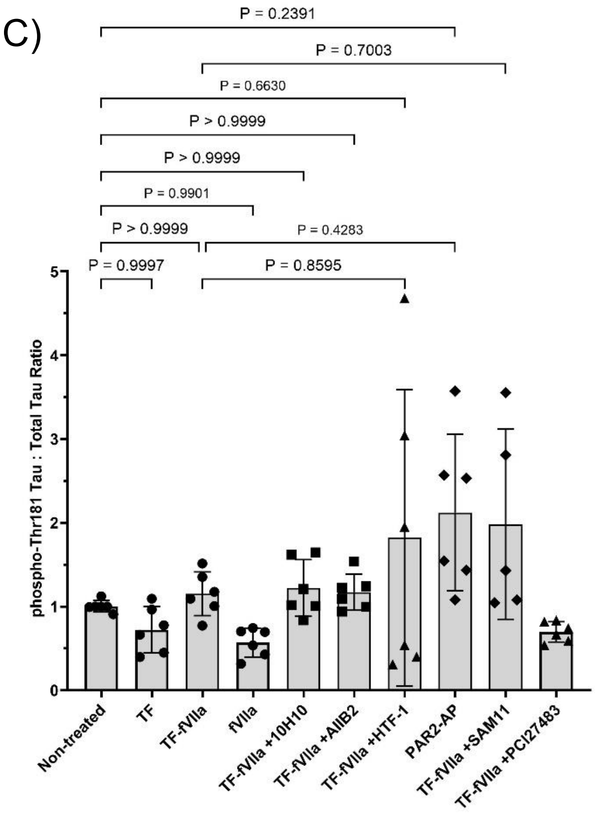

A) SH-SY5Y and B) HCN-2 cells (2 × 10^5) were treated with recombinant relipidated Innovin TF (0.65 ng/ml) as described in figure 4 in the main text. Sets of

cells were harvested at 24 h. Another set of SH-SY5Y and HCN-2 cells were repeatedly supplemented and harvested at 72 h. C) Sets of SH-SY5Y cells were treated as detailed in figure 5 and harvested after 24 h. All samples were analysed by western blot, with rabbit anti-human Tau, or rabbit anti-human Tau phospho-Thr181 antibodies and then developed with goat anti-rabbit IgG alkaline phosphatase-conjugated antibody. Band densities were determined using the ImgaeJ software, normalised against the respective GAPDH, and then, the ratios of phospho-Tau to Tau protein was calculated. Presented data include the calculated mean values ± the calculated standard deviation, as in the original figures.

Supplementary Figure 5: Calculated ratios of phospho-Tau : Total Tau following treatment with TF or TF-fVIIa

A)

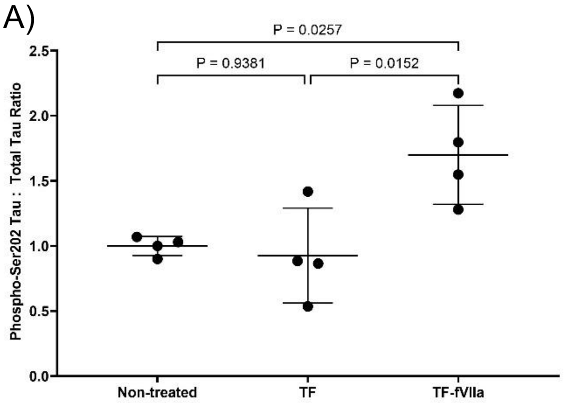

B)

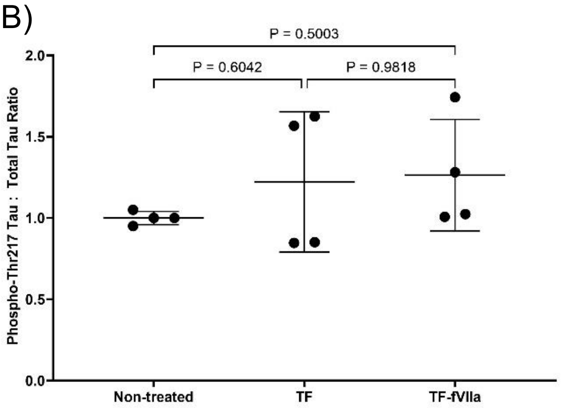

C)

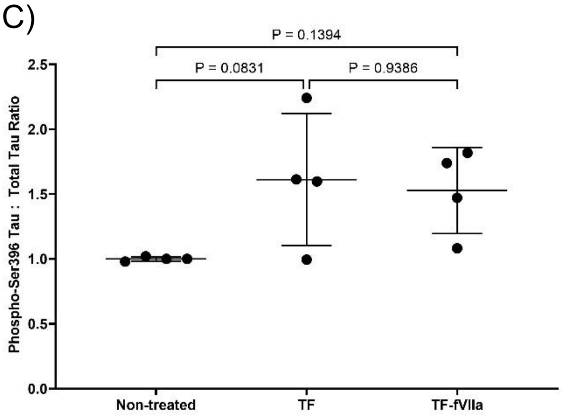

D)

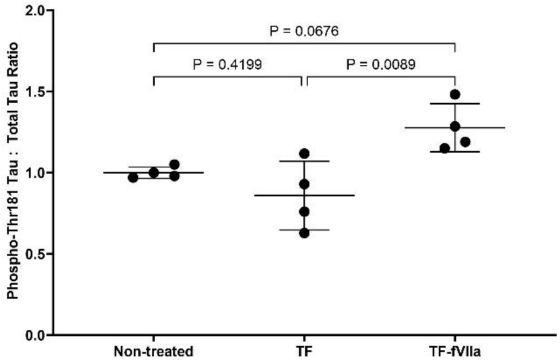

E)

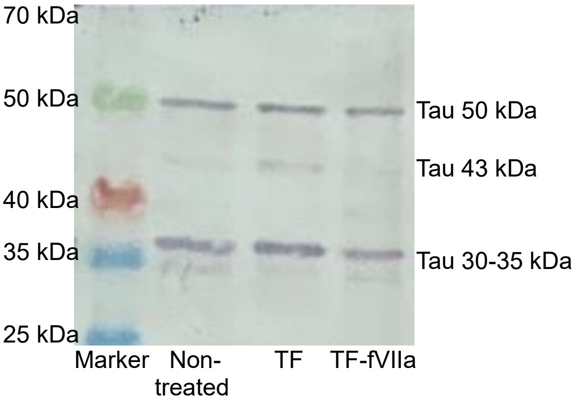

SH-SY5Y (2 × 10^5) were treated with recombinant relipidated Innovin TF (0.65 ng/ml) together with or without human fVIIa (5 nM). Cells were harvested at 24 h and analysed by western blot and probed with A) rabbit anti-human phospho-Ser202 Tau antibody, B) rabbit anti-human phospho-Thr217 Tau antibody, C) rabbit anti-human phospho-Ser396 Tau antibody, D) rabbit anti-human phospho-Thr181 Tau antibody, as

well as with E) rabbit anti-human Tau antibody as described in figure 6. Membranes were then developed with goat anti-rabbit IgG alkaline phosphatase-conjugated antibody. Band densities were determined using the ImgaeJ software, normalised against the respective GAPDH and then the ratios of phospho-Tau to the relevant Tau protein band was calculated. Presented data include the calculated mean values ± the calculated standard deviation, from 4 biological experiments.

Supplementary Figure 6: Time-course analysis of the Tau protein fragments in differentiated SH-SY5Y cells, following treatment with recombinant TF

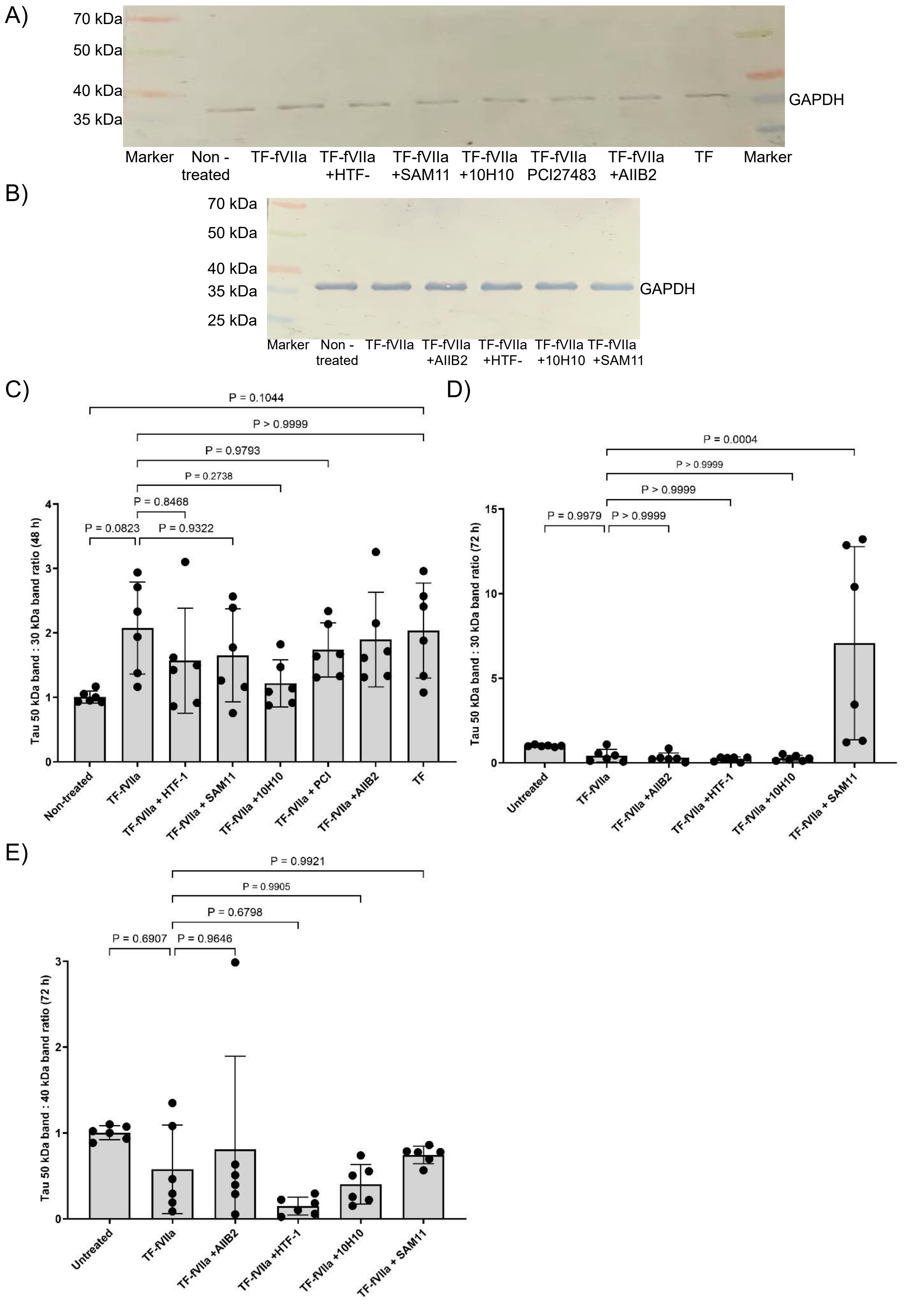

SH-SY5Y (2×10^5) were treated with various reagents as indicated in each column and described in figure 8. The cells were harvested at 48 h and 72 h, post-treatment and were analysed by western blot described in figure 8. The samples were also probed with goat anti-human GAPDH at A) 48 h and B) 72 h, post-treatment and developed using a donkey anti-goat IgG alkaline phosphatase-conjugated antibody. The bands were visualised, and band densities were analysed using ImageJ software. The data from figure 8 were used to calculate the ratio of 50 kDa : 30 kDa bands at C) 48 h and D) at 72 h, and E) to calculate the ratio of 50 kDa : 40 kDa band at 72 h, post-treatment.

Supplementary Figure 7: Fluorescence analysis of the formation of aggregates in response to TF.

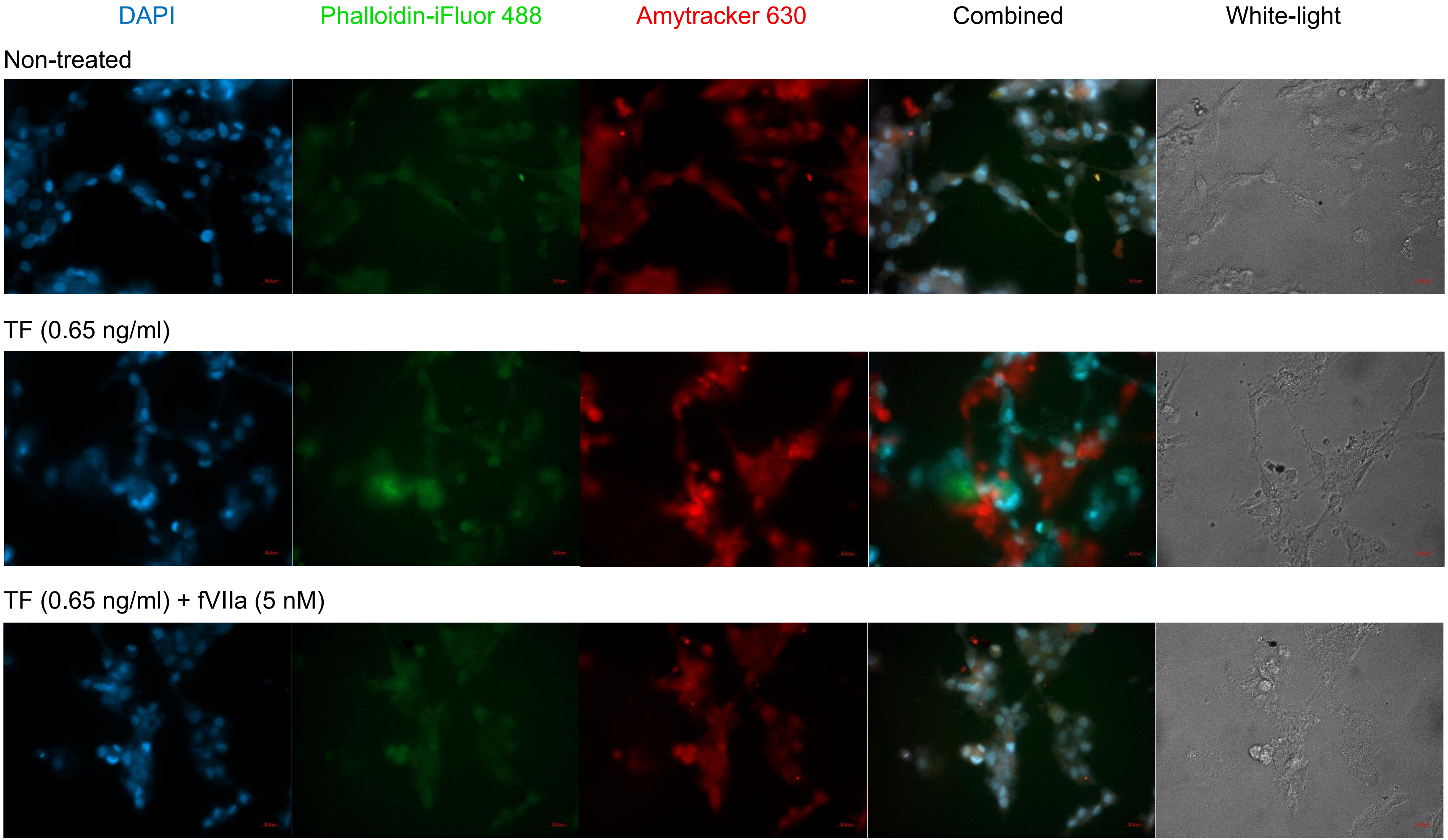

SH-SY5Y were plated in 29 mm culture dishes with a 10 mm glass bottomed micro-well and differentiated as above. Aliquots of the differentiated cells were treated daily with TF (0.65 ng/ml) in the presence and absence of fVIIa (5 nM) for up to 3 days. The cells were then fixed, washed and probed with Amytracker 630 (1 µg/ml) in distilled water. The cells were stained with DAPI (2 µg/ml) and Phalloidin-iFluor 488 (2 µg/ml). Images were acquired at room temperature, using a Zeiss Axio Vert.A1 inverted fluorescence microscope at × 40 magnification and acquired using the ZEN software.

Supplementary Figure 8: Examination of the release of Tau into the media, in response to TF

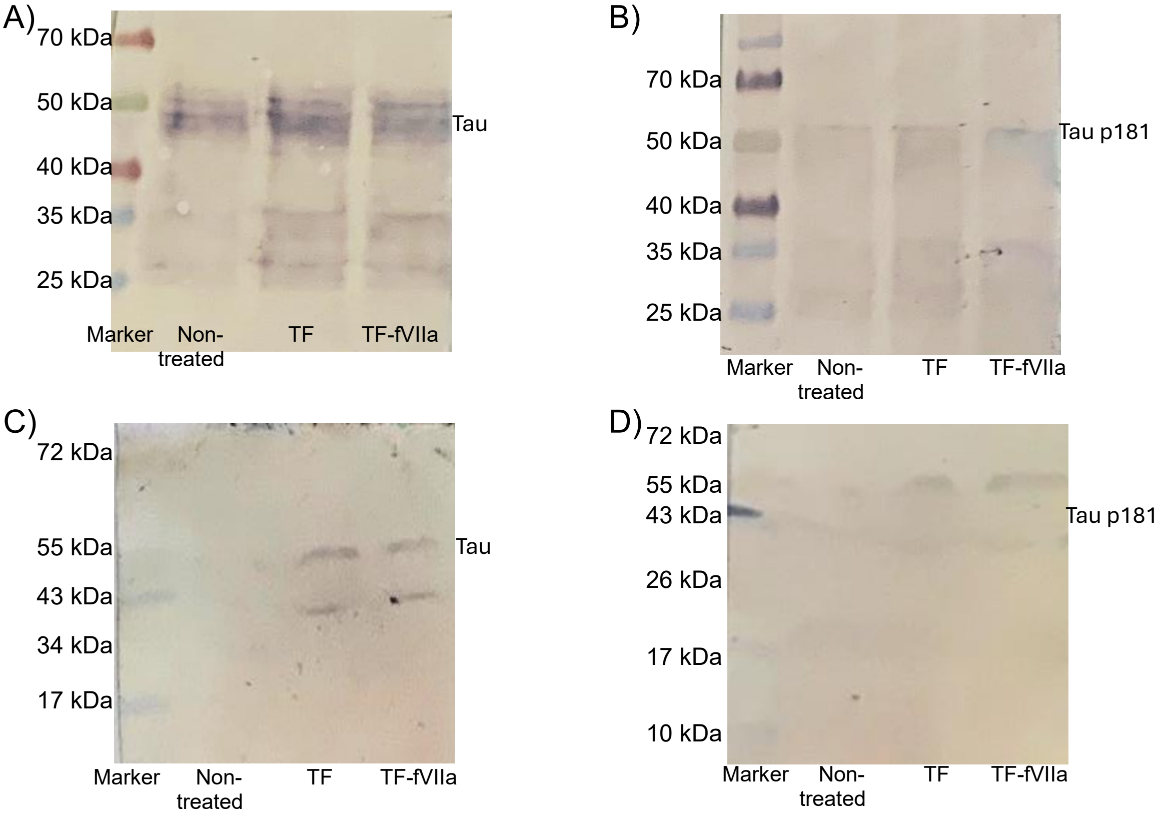

SH-SY5Y cells (2 × 10^5; A and B) and HCN-2 cells (2 × 10^5; C and D) were repeatedly treated with recombinant relipidated Innovin TF (0.65 ng/ml) in the presence and absence of human fVIIa (5 nM). The conditioned media were collected at 72 h and centrifuged at 3,000 *g* for 5 mins to remove cell debris. The proteins were then concentrated using a Centricon concentrators with 3 kDa cutoff and centrifuged at 3,000 *g* for 5 h, at 4°C. The retained proteins were then separated by denaturing 12% (w/v) polyacrylamide gel electrophoresis, transferred to a nitrocellulose membrane and probed with a rabbit anti-human Tau antibody (A and C), or a rabbit anti-human Tau phospho-Thr181 antibody (B and D) diluted 1:3,000 (v/v) in TBST. The membranes were then developed with goat anti-rabbit IgG alkaline phosphatase-conjugated antibody. The bands were visualised using the Western Blue stabilised

alkaline phosphatase-substrate and bands recorded. (Images are representative of 3 separate experiments).
